# Structural, Electronic, and Magnetic Properties of Neutral Borometallic Molecular Wheel Clusters

**DOI:** 10.3390/ma18020459

**Published:** 2025-01-20

**Authors:** Saira Perveen, Nevill Gonzalez Szwacki

**Affiliations:** Faculty of Physics, University of Warsaw, Pasteura 5, PL-02093 Warsaw, Poland; sperveen@fuw.edu.pl

**Keywords:** borometallic clusters, planar boron clusters, first-principles calculations, nanostructure design

## Abstract

Atomic clusters exhibit properties that fall between those found for individual atoms and bulk solids. Small boron clusters exhibit planar and quasiplanar structures, which are novel materials envisioned to serve as a platform for designing nanodevices and materials with unique physical and chemical properties. Through past research advancements, experimentalists demonstrated the successful incorporation of transition metals within planar boron rings. In our study, we used first-principles calculations to examine the structure and properties of neutral boron clusters doped with transition metals, denoted as TMB_*n*_ and TMB_2*n*_, where TM = Ti, Cr, Mn, Fe, Co, Nb, or Mo and n=8–10. Our calculations show that the TMB_2*n*_ structures, which involve sandwiching metal atoms between two rings (called the drum configuration), and clusters with the single ring configuration, TMB_*n*_, are stable. These clusters typically have relatively large HOMO-LUMO energy gaps, suggesting high kinetic stability and low chemical reactivity. Moreover, the clusters display interesting magnetic properties, determined not only by the metal atoms but also by the induced magnetism of the boron rings. These structures have potential applications in spintronics and sensing. This work also provides a basis for studying magnetism in the one-dimensional limit.

## 1. Introduction

Boron, a versatile element with applications spanning many fields, has a rich history that traces its origins to the isolation of simple boranes by Stock et al. [1]. Over the past two decades, a noteworthy chapter in this narrative has unfolded with the synthesis and characterization of various low-dimensional boron nanomaterials, including nanoclusters, nanowires, nanotubes, nanobelts, nanoribbons, and monolayer crystalline sheets [2,3,4,5]. Unlike traditional bulk boron crystals found in α-, β-, and γ-rhombohedral and α-tetragonal forms, these newly crafted boron-based nanomaterials have distinctive bonding patterns [6]. This departure gives rise to captivating physical and chemical properties, making them a focal point of fascination within materials science. Of particular note is the capability of boron-based nanomaterials, such as clusters, to serve as superatoms or as fundamental building blocks for crafting nanostructures designed with novel functionalities and properties. These systems are characterized by having diverse structures, including planar, quasiplanar, bowl, cage, tubular drum-like forms, multiple ring tubes, and fullerenes [7,8,9,10,11]. Electron deficiency of the boron atom enables its involvement in localized and delocalized bonding patterns across various geometric configurations. The neutral B_*n*_ clusters with n<20 prefer a planar or quasiplanar structure [12]. On the other hand, $B_{n}^{-}$ clusters retain planarity (or quasiplanarity) for n≤40 [13]. The perfectly planar B_8_ and $B_{9}^{-}$ molecular wheels, with a hepta- or octacoordinated central boron atom, respectively, laid the foundation of borometallic clusters [14]. In the borometallic cluster, the central B atom is replaced by a metal atom, creating a similar doubly aromatic cluster [15,16]. Experiments in cluster beams have demonstrated that transition metal (TM) atoms can be incorporated into the center of the planar boron clusters [17,18,19].

In a more recent study by Zhang et al. [20], the CALYPSO structure search method was used to investigate the global minimum structures of neutral and negatively charged Ta_2_B_*n*_ clusters, where *n* ranges from 1 to 10. Their findings revealed the formation of a B ring when *n* equals 6. Interestingly, the research identified a neutral Ta_2_B_6_ cluster exhibiting a bipyramidal configuration with enhanced stability. Chang Xu et al. [21] conducted a theoretical investigation on TM-centered double-ring clusters, M@B_2*n*_ (where M = Ti, Cr, Fe, Ni, Zn and *n* = 6, 7, 8). They explored the factors contributing to the stability of these clusters in their study. Miao Yan et al. [22] introduced the concept of fluxional bonds in various structures, including planar $B_{19}^{-}$, tubular Ta@$B_{20}^{-}$, and cage-like $B_{39}^{-}$. These bonds form and break continuously on these systems’ extremely flat potential energy surfaces. Ren et al. [23] used the CALYPSO method combined with density functional theory (DFT) calculations on niobium-doped boron clusters. Their research revealed that the global minima for the neutral clusters correspond to half-sandwich structures at n=10–17 and tubular-type structures at n=18–20. Chen et al.’s [24] study offered a comprehensive overview of the geometric configurations of both pure (B_*n*_) and metal-doped boron clusters, which exhibit structures such as planar, nanotube, bilayer, fullerene-like, and core-shell forms across a wide range of cluster sizes (*n*). They also explored the potential for developing boron-based nanomaterials with specific functionalities derived from metal–boron clusters. Liu et al. [25] identified ten potential clusters made up of rings formed by 12 to 15 atoms of boron–carbon doped with heavier alkaline-earth (Ae) metals (Ae = Ca, Sr, Ba). Their chemical bond analysis reveals the important role of Ae *d* orbitals in facilitating covalent interactions between the central Ae atom and the surrounding boron–carbon rings.

Linear magnetic chains are becoming crucial in quantum information involving entanglement and correlation [26,27]. Carbon and boron-nitride-based nanotubes are used as support surfaces to stabilize unstable isolated atomic chains [28]. Photoelectron spectroscopy and theoretical studies identified metal-centered tubular ionic structures ${\mathrm{MnB}}_{16}^{-}$, ${\mathrm{CoB}}_{16}^{-}$, ${\mathrm{RhB}}_{18}^{-}$, and ${\mathrm{TaB}}_{20}^{-}$ with a magnetic moment of 2, 2, 0, and 0 μB, respectively [29,30,31,32]. Neutral boron-based tubular structures, including FeB_14_, FeB_16_, UB_20_, NpB_20_, and PuB_20_, were theoretically examined and found to have a quenched magnetic moment of 2 μB for the iron-based structures and relatively high magnetic moment of 3.69, 4.92, and 6.07 μB, respectively, for actinide-centered tubular structures [33,34]. These research works showed that the size and stability of the tubular structures greatly depend on the encapsulated metal atoms through the covalent bonds forming between the metal ions and the boron frame (MB_*n*_) [35]. Metal-centered tubular boron clusters have been suggested as the building blocks for one-dimensional metallo-nanotubes [35]. However, the magnetic moments of TM-centered tubular boron clusters remain unexplored, with limited discussion on their magnetic behaviors.

In this work, using DFT calculations, we study the structure and properties of molecular borometallic wheel structures derived from planar boron B_8_, B_9_, and B_10_ wheel clusters. For all of these clusters, we analyze their magnetic behavior using spin-polarized calculations and analyze charge density differences and the contribution of metal and boron atoms to the density of states via the projected density of states (PDOS) analysis.

## 2. Computational Details

**Figure 1:**
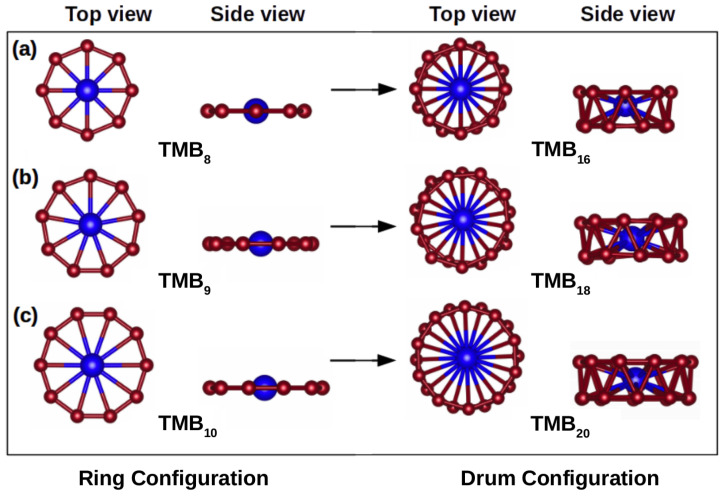
The structure of TMB_*n*_ (**left**) and TMB_2*n*_ (**right**) for n=8, 9, and 10 in (**a**–**c**), respectively. In each case, top and side views of the clusters are shown.

In our DFT-based study, we use the plane-wave-based Quantum ESPRESSO package [36]. The generalized gradient approximation (GGA) of Perdew–Burke–Ernzerhof (PBE) [37] is employed in the calculations combined with projector-augmented wave (PAW) pseudopotentials. Boron-based rings centered with TM atoms (TMB_*n*_) are modeled by varying the value of *n* from 8 to 10 for single rings and subsequently increasing it to 16, 18, and 20 by taking the same ring twice and sandwiching the metal atom between them (TMB_2*n*_). The TMB_*n*_ and TMB_2*n*_ clusters are shown in Figure 1. A vacuum distance of 16 Å is introduced to avoid interactions between cluster replicas. A plane-wave basis set with an energy cutoff of 70 Ry is used. The convergence threshold for the self-consistent energy calculations is $10^{-12}$, and the atomic positions are optimized until the forces on atoms are smaller than $10^{-4}$ Ry/Bohr. The convergence threshold during phonon self-consistent calculations is $10^{-14}$ eV. All the computations are carried out at the Γ-point of the Brillouin zone. Assessing the stability of clusters often relies on the binding energy, $E_{b}$, as an important parameter. The calculation of $E_{b}$ for the TMB_*n*_ and TMB_2*n*_ clusters is conducted using the following equation:

$$E_{b}=E\left({\mathrm{TMB}}_{i}\right)-E\left(B_{i}\right)-E\left(\mathrm{TM}\right),$$

where $E({\mathrm{TMB}}_{i})$ is the total energy of the optimized boron ring with the TM atom in the center, $E(B_{i})$ is the total energy of the boron cluster in the ring (i=n) or drum (i=2n) forms, and E(TM) is the energy of the isolated TM atom. Spin-polarized calculations are carried out to study the magnetic properties of the clusters.

## 3. Results and Discussion

We became interested in the high symmetry of aromatic boron B_8_ and $B_{9}^{-}$ clusters, which are perfect heptagon and octagon structures with D_7*h*_ and D_8*h*_ symmetries, respectively [38]. This led us to conduct thorough investigations involving neutral clusters formed by replacing the central B atom in the octagon boron structure with TM counterparts, specifically TM = Ti, Cr, Mn, Fe, Co, Nb, and Mo, and extending the study to larger borometallic clusters. Our investigation focused on two primary configurations: the above-mentioned “ring configuration” (TMB_*n*_), featuring a TM atom at the center of a ring composed of 8, 9, or 10 boron atoms, and the “drum configuration” (TMB_2*n*_), involving two rings of the same size stacked on top of each other, with the metal atom situated between the centers of two rings comprising 8, 9, or 10 boron atoms. Our system is visually represented in Figure 1, with boron atoms highlighted in dark red and forming a ring, and the central TM atom is shown in blue.

### 3.1. Structural Properties

In line with the structural arrangement shown in Figure 1, the bond lengths between adjacent boron atoms ($d_{B-B}$) and those connecting boron with central metal atoms ($d_{B-\mathrm{TM}}$) are summarized for all the studied clusters in Table 1. It is worth noting that all the designed configurations are planar except for TiB_8_, TiB_9_, and FeB_8_ where the central atom deviates slightly out of plane by 0.8387 Å, 0.6168 Å, and 0.8394 Å, respectively, giving rise to a distinctive bowl-like structure, which aligns well with previous research. The distances between metal and boron atoms ($d_{B-\mathrm{TM}}$) for n=8, 9, and 16 are small compared to their covalent bonds, indicating minimal steric repulsion between them. The bond length $d_{B-B}$ is influenced by the number of B atoms present in the boron ring and the size of the central metal atom, especially for n=8 and 9 (refer to Table 1). For TM = Cr, Mn, Fe, or Co, the drum configuration with the maximum number of boron atoms is only possible with B_18_ because, beyond that limit, an elliptical geometry prevails over a circular shape. In Table 1, we summarize the point group (PG) symmetry for each cluster, which was determined using the AFLOW software [39]. Except for the non-planar structures, the $D_{4h}$ symmetry is assigned to TMB_8_. For TMB_10_ clusters, the PG symmetry is $D_{2h}$ due to its nearly circular geometry or $C_{2h}$ due to its elliptical geometry. Similar trends have been reported for ${\mathrm{TaB}}_{n}^{-}$ clusters [40]: ${\mathrm{TaB}}_{8}^{-}$ forms a pyramidal structure, ${\mathrm{TaB}}_{9}^{-}$ shows increased planarity as the Ta atom integrates into a larger boron ring, and ${\mathrm{TaB}}_{10}^{-}$ achieves a perfectly planar, decacoordinated $D_{10h}$ symmetry, representing the highest coordination for Ta in planar geometry, stabilized by aromaticity. Drum configurations mostly belong to the $C_{1}$ PG symmetry.

In Table 1, we have summarized the values of $E_{b}$ for all the clusters. The $E_{b}$ values consistently increase from TMB_8_ to TMB_9_ (or, in some cases, TMB_8_ to TMB_10_) and then decrease gradually until reaching TMB_20_ across all seven systems for each TM. Overall, the $E_{b}$ values are relatively high for Co-doped configurations but notably lower for Mn-doped structures. It is important to note that all structures have negative $E_{b}$ values, indicating the stability of the clusters since energy is required to separate them into TM and B_*n*_.

### 3.2. Electronic Properties

In Table 1, we have summarized the HOMO-LUMO (H-L) energy gaps of the studied clusters. Although DFT-GGA underestimates energy gap values, most clusters still exhibit nonzero H-L energy gaps. The largest energy gaps for the ring configurations are found in CoB_9_, NbB_10_, and FeB_9_, with values of 1.055 eV, 0.907 eV, and 0.785 eV, respectively. Among the drum configurations, the H-L energy gaps for MoB_20_, CrB_16_, and MnB_16_ (1.088 eV, 0.822 eV, and 0.804 eV, respectively) are larger compared to other drum structures. It is well established that DFT-based approaches systematically underestimate energy gaps [41]. Therefore, the true H-L energy gaps of the clusters may be even twice larger. Structures with large H-L energy gaps are associated with high kinetic stability and low chemical reactivity, making them promising candidates for future applications.

The PDOS spectra for all the clusters can be found in Section SI of the Supplementary Materials (SM). These spectra are valuable for studying the contribution of different atomic orbitals to the density of states in the studied clusters, particularly at and near the Fermi level, $E_{F}$. They also aid in visualizing the contribution of atomic orbitals to the magnetic properties of the clusters (e.g., to the shift between the spin-up and spin-down PDOS). In general, the *p*-orbitals of boron and the *d*-orbitals of TMs make the most significant contributions to the density of states at the $E_{F}$. However, in specific cases such as TiB_9_, FeB_8_, CoB_8_, and NbB_20_, the contribution at the $E_{F}$ comes solely from boron *p*-orbitals. A combination of boron *p* and TM *d* orbitals contribute to the density of states at the $E_{F}$ for CrB_8_, CrB_18_, FeB_16_, MoB_9_, and MoB_10_.

Cluster-specific observations, as inferred from the PDOS spectra, show distinct contributions to the highest occupied states, which can be summarized as follows: In the Ti series, B *p*-orbitals dominate in TiB_9_, TiB_10_, and TiB_20_. In contrast, a mix of B *p* and TM *d* occurs in TiB_8_, TiB_16_, and TiB_18_. In the Cr series, B *p*-orbitals significantly contribute in CrB_9_ and CrB_16_, and a mix of B *p* and TM *d* contributions occur in the rest of the Cr-based clusters. In the Mn and Fe series, the clusters frequently exhibit combined B *p* and TM *d* contributions with a consistent magnetic character. For Co-based clusters, the electronic configurations reveal that boron *p*-orbitals dominate the highest occupied states in CoB_8_, CoB_16_, CoB_18_, and CoB_20_, all of which are magnetic except for CoB_8_, which lacks an H-L energy gap. In contrast, CoB_9_ and CoB_10_ exhibit contributions from both boron *p*- and TM *d*-orbitals to the highest occupied states, with CoB_9_ being nonmagnetic and CoB_10_ magnetic. Similarly, in Nb-based clusters, boron *p*-orbitals primarily contribute to the highest occupied states in NbB_8_, NbB_16_, and NbB_18_, which are magnetic with H-L energy gaps, and NbB_20_, which is nonmagnetic without an H-L energy gap. Clusters NbB_9_ and NbB_10_ exhibit combined contributions from boron *p*- and TM *d*-orbitals, maintaining magnetic properties and distinct H-L energy gaps. Finally, in the Mo series, most of the structures are nonmagnetic (e.g., MoB_18_ and MoB_20_), which is reflected in the symmetry of the spin-up and spin-down PDOS, contrasting with the magnetic nature of smaller clusters like MoB_9_.

These observations highlight the nuanced role of TM *d*-orbitals and B *p*-orbitals in shaping the electronic and magnetic characteristics of borometallic clusters. The variability in orbital contributions underscores the tunable properties of these materials.

### 3.3. Vibrational Properties

The vibrational properties of the structures were analyzed using phonon computations to assess their stability. These calculations provide valuable insights into the dynamic behavior and structural integrity of the systems under study. According to the literature [42], a cluster is considered stable if all its real vibrational frequencies are positive and the minimum vibrational frequency, denoted as $f_{min}$, is significantly large. Table 2 presents the values of $f_{min}$ for each cluster. For the ring configuration, TMB_8_ and TMB_9_ with TM = Ti, Cr, Mn, Fe, or Co have the highest values of $f_{min}$, whereas TMB_9_ and TMB_10_ clusters have the highest $f_{min}$ for TM = Nb and Mo. Interestingly, the FeB_10_ cluster in our calculations is at the border of stability as its $f_{min}$ value is small but negative. For the drum configurations, there is less regularity, and large values of $f_{min}$ are obtained for CrB_16_, while the lowest value is for MnB_20_. Our results for $f_{min}$ are in close agreement with a study by Pu et al. [19], where the values of $f_{min}$ for FeB_9_ and CoB_9_ were 110.5 cm^−1^ and 96.7 cm^−1^, respectively. The complete list of phonon frequencies for each cluster is provided in Section SII of SM.

### 3.4. Dynamic Properties

**Figure 2:**
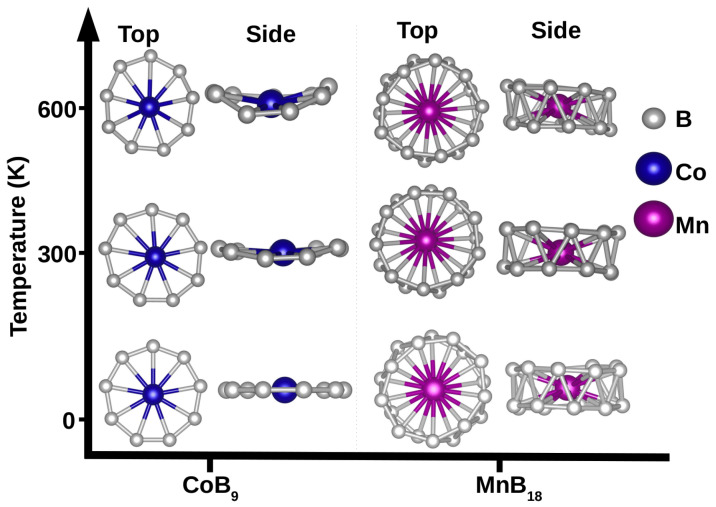
Ab initio molecular dynamics simulations at 300 K and 600 K. The top and side views of CoB_9_ and MnB_18_ clusters are shown for each temperature.

To further verify the stability of the ring and drum structures, we performed quantum molecular dynamics (MD) simulations at two different temperatures with the choice of 1 fs for the time step. Each run was 3.5 ps long. At a temperature of 300 K, the ring and drum structures maintain their shape during the MD run, and no significant deformations occur. Both structure types deform at a temperature of 600 K but retain their wheel-like structure. This is shown in Figure 2 on the example of CoB_9_ and MnB_18_. It is worth noting that MnB_18_ has one of the smallest $E_{b}$ in absolute value.

### 3.5. Magnetic Properties

The magnetic moment values, denoted as *m*, for each cluster, are listed in Table 1. Generally, the value of *m* decreases as the number of boron atoms in the ring increases from 8 to 10. In contrast, an opposite trend is observed for drum structures, with *m* tending to increase as the number of boron atoms in the TMB_*n*_ structure increases (from 16 to 20). Interestingly, the *m* values for CoB_*n*_ and MoB_*n*_ drum structures are independent of *n*, remaining equal to 1 μB and 0 μB, respectively.

**Figure 3:**
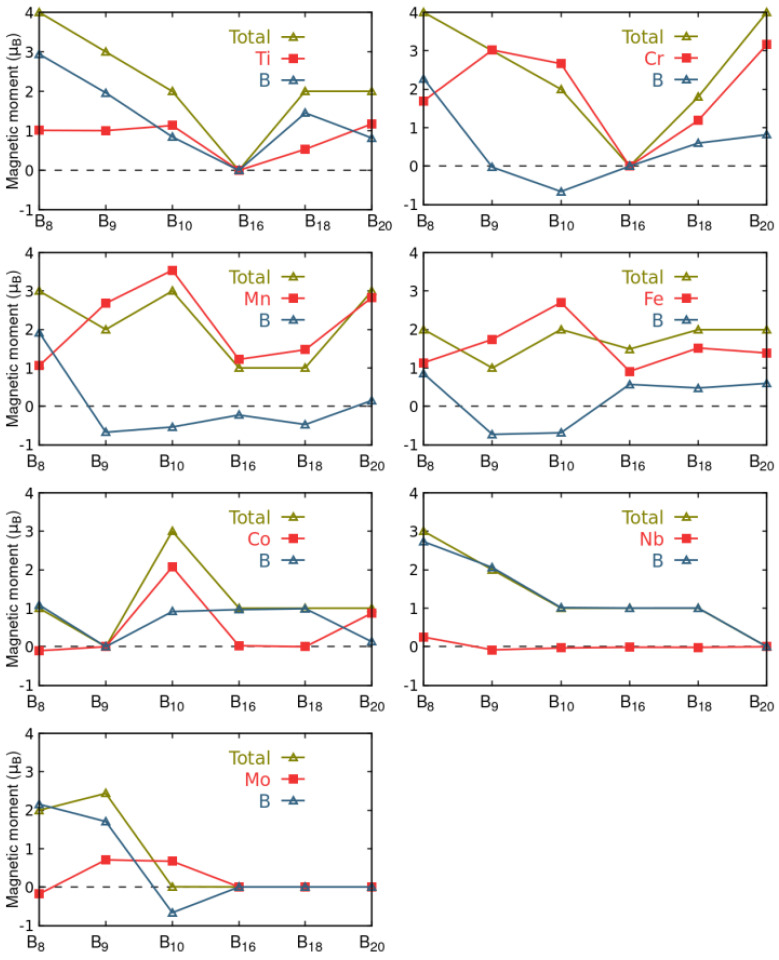
Total magnetic moment *m* and local magnetic moment contributions on the *y*-axis versus the cluster type in the *x*-axis. The contributions come from the TM (TM = Ti, Cr, Mn, Fe, Co, Nb, or Mn) atom and the boron atoms of each TMB_*n*_ cluster. In the graph, the total magnetization is represented by yellow triangles, the magnetization due to boron atoms is represented by blue triangles, and the red squares represent the contribution from the TMs. The lines are guides for the eye.

The contribution from boron and TM atoms to magnetization is illustrated in Figure 3. In general, the contribution from TM atoms to total *m* is large. However, in most cases, the boron atoms become polarized, resulting in a non-zero contribution to *m*. Specifically, for TMB_8_ clusters, the boron contribution (represented by the yellow line) to *m* is consistently larger than the TM contribution (represented by the blue line). Conversely, metals demonstrate dominance in the case of TMB_10_ structures. For drum configurations, both metal and boron atoms consistently exhibit positive (or zero) contributions to *m*, except for the case of Mn, where boron atoms have slightly negative local magnetization. The case of NbB_*n*_ clusters is particularly interesting, as TM atoms induce the magnetization, but *m* is entirely localized on the boron atoms.

The result presented in Figure 3 can be spatially visualized by drawing the difference between the spin-up charge density ($\rho^{↑}$) and the spin-down charge density ($\rho^{↓}$):

$$\mathrm{spin}\mathrm{polarization}\mathrm{density}=\rho^{↑}\left(\vec{r}\right)-\rho^{↓}\left(\vec{r}\right).$$

**Figure 4:**
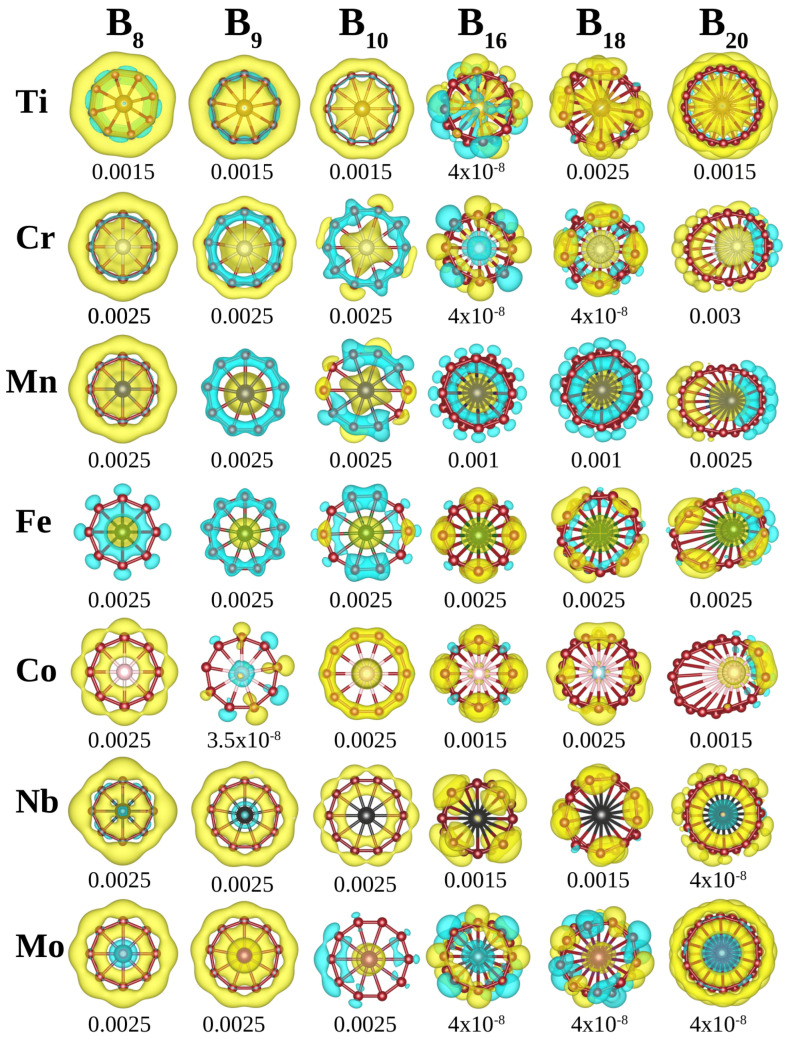
Difference between the spin-up charge density and the spin-down charge density. Positive and negative values are represented with yellow and cyan, respectively. The value of the iso-surface is given below each system.

This can be seen in Figure 4 where the positive and negative values of the spin polarization density are represented with yellow and cyan, respectively. This figure clearly shows that the boron atoms in all clusters become polarized, and this polarization significantly influences the total value of *m*. Our Löwdin population analysis provided in Section SIII of SM quantitatively demonstrates this. This analysis evidenced, in most cases, a charge transfer from/to the TM atom, which gives rise to polarization effects on the boron atoms that, as a result, contribute to the total *m*. The results suggest that the polarization of boron atoms is important in modulating the electron distribution, which may affect the cluster’s reactivity and interactions in different environments.

## 4. Summary and Conclusions

The successful integration of TM atoms into the core of planar boron clusters B_*n*_ creates a fascinating class of borometallic molecules. In our study, we present the results of first-principles calculations aimed at examining the structural, electronic, and magnetic properties of boron rings with a TM atom in the center, TMB_*n*_ (with TM being Ti, Cr, Mn, Fe, Co, Nb, or Mo, and *n* being 8, 9, or 10), as well as boron double-rings sandwiching the metal atom, TMB_2*n*_. In these molecular structures, each B atom in the circumference provides two electrons to the B–B peripheral covalent bonds and one electron to the delocalized B–TM bonds. Consequently, a TM atom with the appropriate number of valence electrons can be accommodated in the center of the boron wheel to form the TMB_*n*_ clusters, resulting in a structurally and dynamically stable borometallic cluster compound. Most of the studied clusters also possess relatively high H-L energy gaps, which may suggest low chemical reactivity. Our investigation also indicates that the overall magnetic moment of the clusters is a combination of the magnetic moment of the central TM atom and the induced magnetic moment of the peripheral boron atoms. These molecular-based magnets provide a platform for studying magnetism in the zero-dimensional limit. Furthermore, the TMB_2*n*_ clusters can be building blocks for one-dimensional tubular forms.

## Figures and Tables

**Table 1:** Calculated parameters related to each studied structure: bond length between boron atoms ($d_{B-B}$), bond length between boron and metal atoms ($d_{B-\mathrm{TM}}$), point group (PG), magnetic moment (*m*), H-L energy gap, and binding energy ($E_{b}$).

${\mathrm{MB}}_{n}$	$d_{B-B}$	$d_{B-\mathrm{TM}}$	PG	*m*	H-L Gap	$E_{b}$
	(Å)	(Å)		($\mu_{B}$)	(eV)	(eV)
TiB_8_	1.578	2.226	$C_{s}$	4	0.541	−9.667
TiB_9_	1.558	2.335	$C_{s}$	3	0.090	−10.692
TiB_10_	1.536	2.485	$D_{2h}$	2	0.617	−11.106
TiB_16_	1.639	2.275	$C_{s}$	0	0.392	−8.214
TiB_18_	1.608	2.460	$C_{1}$	2	0.246	−9.245
TiB_20_	1.579	2.593	$C_{1}$	2	0.312	−7.925
CrB_8_	1.587	2.073	$D_{4h}$	4	0.004	−11.476
CrB_9_	1.551	2.269	$C_{s}$	3	0.367	−12.495
CrB_10_	1.524	2.466	$C_{2h}$	2	0.357	−12.152
CrB_16_	1.614	2.209	$C_{2v}$	0	0.822	−11.234
CrB_18_	1.588	2.432	$C_{1}$	1.8	0.122	−10.471
CrB_20_	1.608	2.645	$C_{1}$	4	0.425	−8.272
MnB_8_	1.572	2.054	$D_{4h}$	3	0.486	−7.288
MnB_9_	1.542	2.255	$C_{2v}$	2	0.880	−7.733
MnB_10_	1.530	2.473	$C_{2h}$	3	0.264	−7.128
MnB_16_	1.602	2.235	$C_{2v}$	1	0.804	−6.472
MnB_18_	1.584	2.431	$C_{1}$	1	0.558	−5.397
MnB_20_	1.602	2.723	$C_{1}$	3	0.439	−3.518
FeB_8_	1.564	2.065	$C_{4v}$	2	0.016	−10.866
FeB_9_	1.533	2.241	$C_{2v}$	1	0.785	−12.050
FeB_10_	1.523	2.461	$C_{2h}$	2	0.274	−11.099
FeB_16_	1.594	2.231	$C_{4v}$	1.49	0.063	−9.955
FeB_18_	1.581	2.445	$C_{1}$	2	0.324	−8.645
FeB_20_	1.617	2.760	$C_{1}$	2	0.268	−7.918
CoB_8_	1.564	2.044	$D_{4h}$	1	0.001	−15.709
CoB_9_	1.539	2.234	$C_{2v}$	0	1.055	−15.889
CoB_10_	1.522	2.462	$D_{2h}$	3	0.404	−14.585
CoB_16_	1.591	2.232	$C_{4v}$	1	0.297	−13.398
CoB_18_	1.579	2.443	$C_{s}$	1	0.311	−11.828
CoB_20_	1.619	2.749	$C_{1}$	1	0.258	−11.881
NbB_8_	1.653	2.157	$D_{4h}$	3	0.504	−10.702
NbB_9_	1.573	2.300	$C_{2v}$	2	0.418	−13.055
NbB_10_	1.533	2.481	$D_{2h}$	1	0.907	−13.451
NbB_16_	1.661	2.306	$C_{s}$	1	0.322	−10.318
NbB_18_	1.561	2.477	$C_{1}$	1	0.362	−12.179
NbB_20_	1.573	2.674	$C_{1}$	0	0.000	−10.937
MoB_8_	1.628	2.126	$D_{4h}$	2	0.419	−12.395
MoB_9_	1.560	2.281	$C_{2v}$	2.44	0.080	−14.021
MoB_10_	1.524	2.467	$C_{s}$	0	0.092	−13.998
MoB_16_	1.658	2.249	$C_{2v}$	0	0.616	−11.811
MoB_18_	1.602	2.431	$C_{1}$	0	0.415	−12.663
MoB_20_	1.568	2.760	$C_{1}$	0	1.088	−10.871

**Table 2:** Minimum vibrational frequencies for each studied cluster.

Ring	$f_{\min}$ (cm^−1^)	Drum	$f_{\min}$ (cm^−1^)
TiB_8_	206.22	TiB_16_	48.41
TiB_9_	123.91	TiB_18_	65.62
TiB_10_	85.00	TiB_20_	78.17
CrB_8_	87.93	CrB_16_	160.53
CrB_9_	196.47	CrB_18_	59.27
CrB_10_	44.03	CrB_20_	89.9
MnB_8_	140.21	MnB_16_	122.13
MnB_9_	135.68	MnB_18_	130.70
MnB_10_	69.60	MnB_20_	27.00
FeB_8_	133.40	FeB_16_	75.14
FeB_9_	128.48	FeB_18_	79.27
FeB_10_	−22.1	FeB_20_	64.30
CoB_8_	132.22	CoB_16_	55.24
CoB_9_	102.07	CoB_18_	70.30
CoB_10_	78.0	CoB_20_	90.34
NbB_8_	45.69	NbB_16_	153.38
NbB_9_	93.35	NbB_18_	38.60
NbB_10_	87.85	NbB_20_	102.61
MoB_8_	29.26	MoB_16_	86.96
MoB_9_	95.29	MoB_18_	41.17
MoB_10_	68.08	MoB_20_	106.48

## Data Availability

The original contributions presented in this study are included in the article. Further inquiries can be directed to the corresponding author.

## Supplementary Materials

The following supporting information can be downloaded at: https://www.mdpi.com/article/10.3390/ma18020459/s1.
