# Supplementary material for: Structural, Electronic, and Magnetic Properties of Neutral Borometallic Molecular Wheel Clusters
Source: Materials (Basel). 2025 Jan 20;18(2):459. doi: 10.3390/ma18020459 (PMC11766989; doi:10.3390/ma18020459)
Supplement: Supplementary file 1 [file materials-18-00459-s001.zip › materials-3401524-supplementary.pdf]

Supporting information for:
Structural, electronic, and magnetic properties of neutral borometallic molecular wheel clusters

Saira Perveen and Nevill Gonzalez Szwacki
Faculty of Physics, University of Warsaw,
Pasteura 5, PL-02093 Warsaw, Poland

CONTENTS

SI. PDOS for each configuration 2

SII. Phonon frequencies for each cluster 9

SIII. Löwdin population analysis 32

SI. PDOS FOR EACH CONFIGURATION

Figures S1 to S7 plot the projected density of states (PDOS) spectra (spin-up and spin-down) for all the studied clusters to visualize the contribution of atomic orbitals to their electronic and magnetic properties.

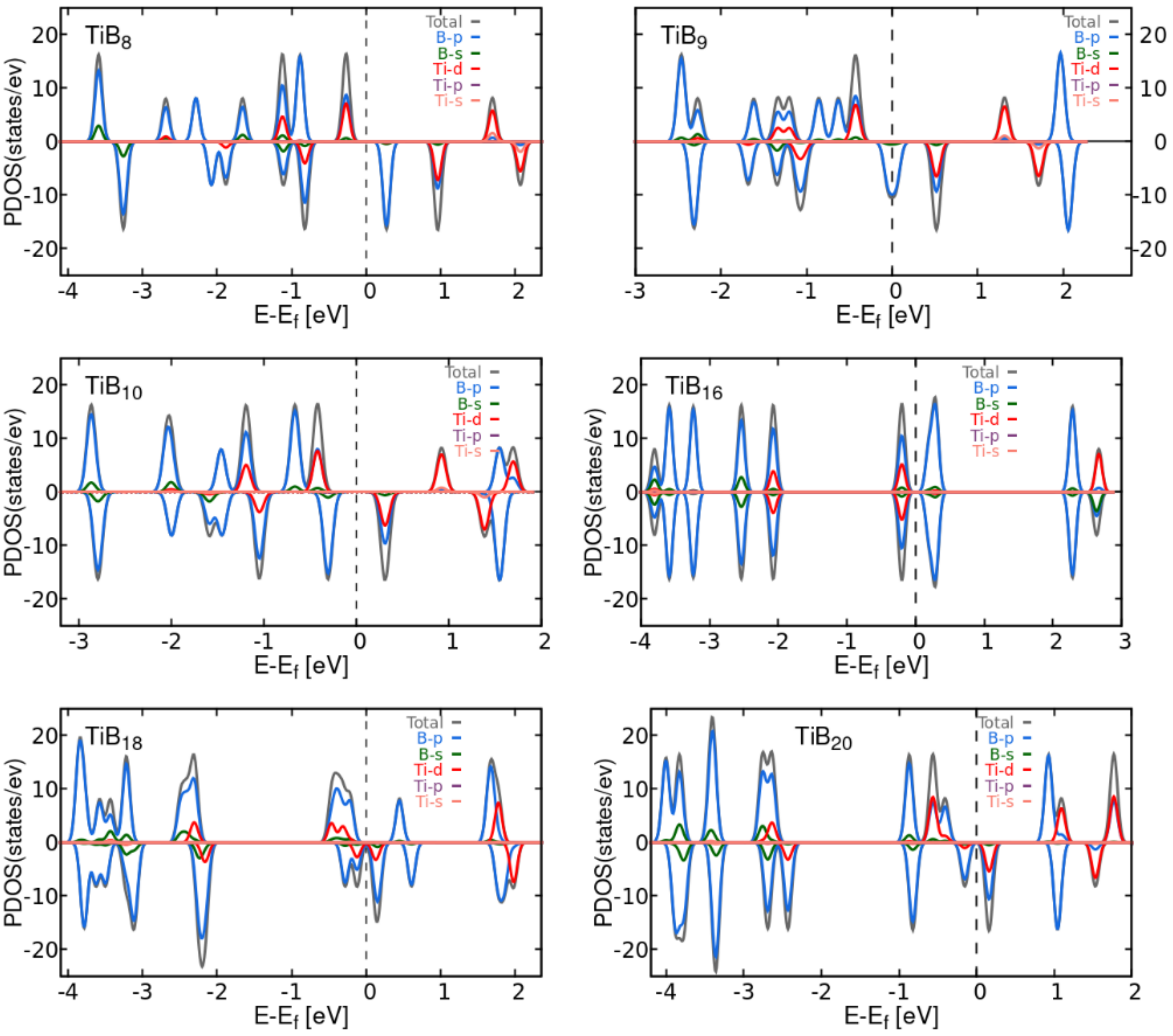

Figure S1. The PDOS for Ti-doped ring and drum structures.

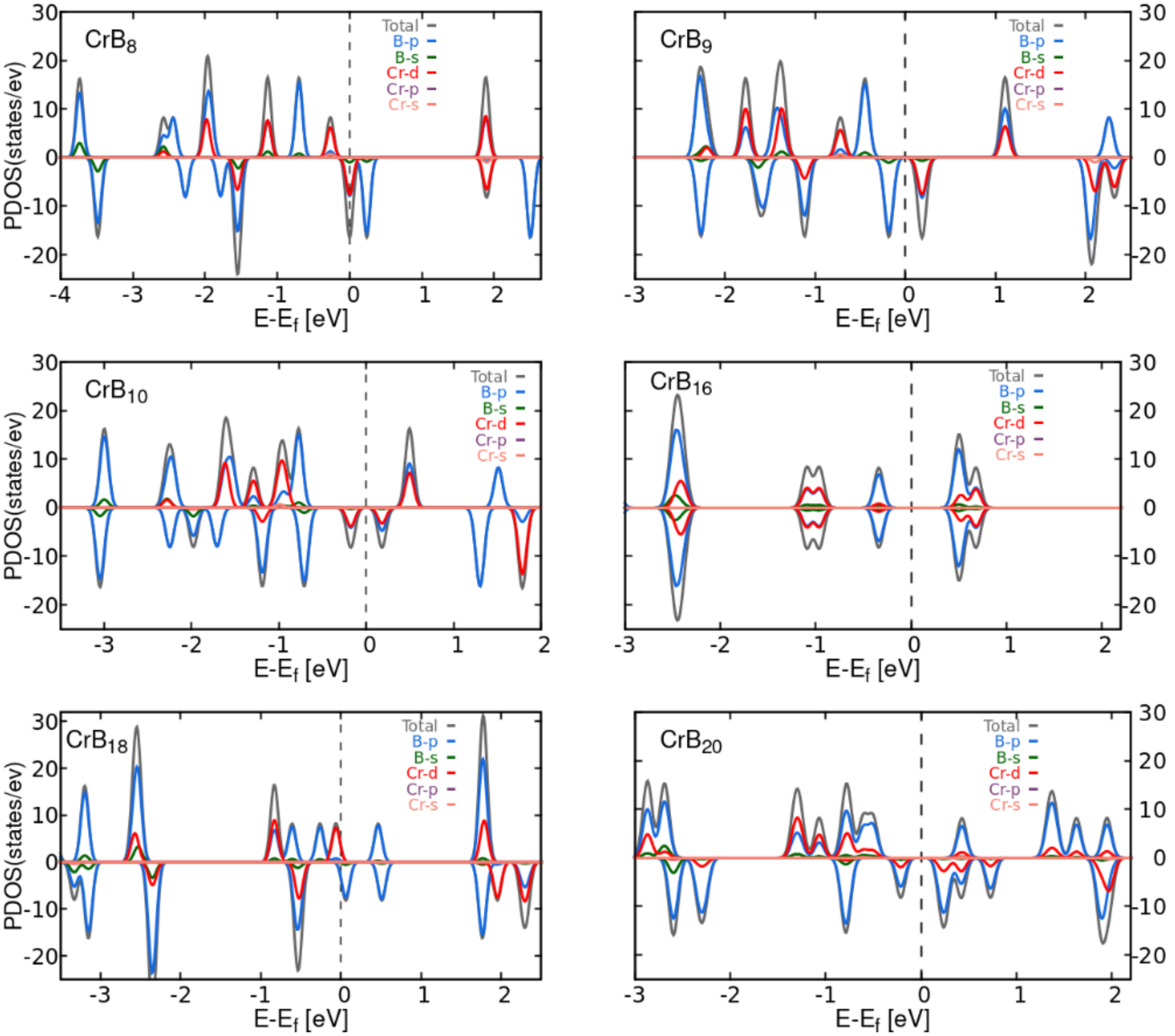

Figure S2. The PDOS for Cr-doped ring and drum structures.

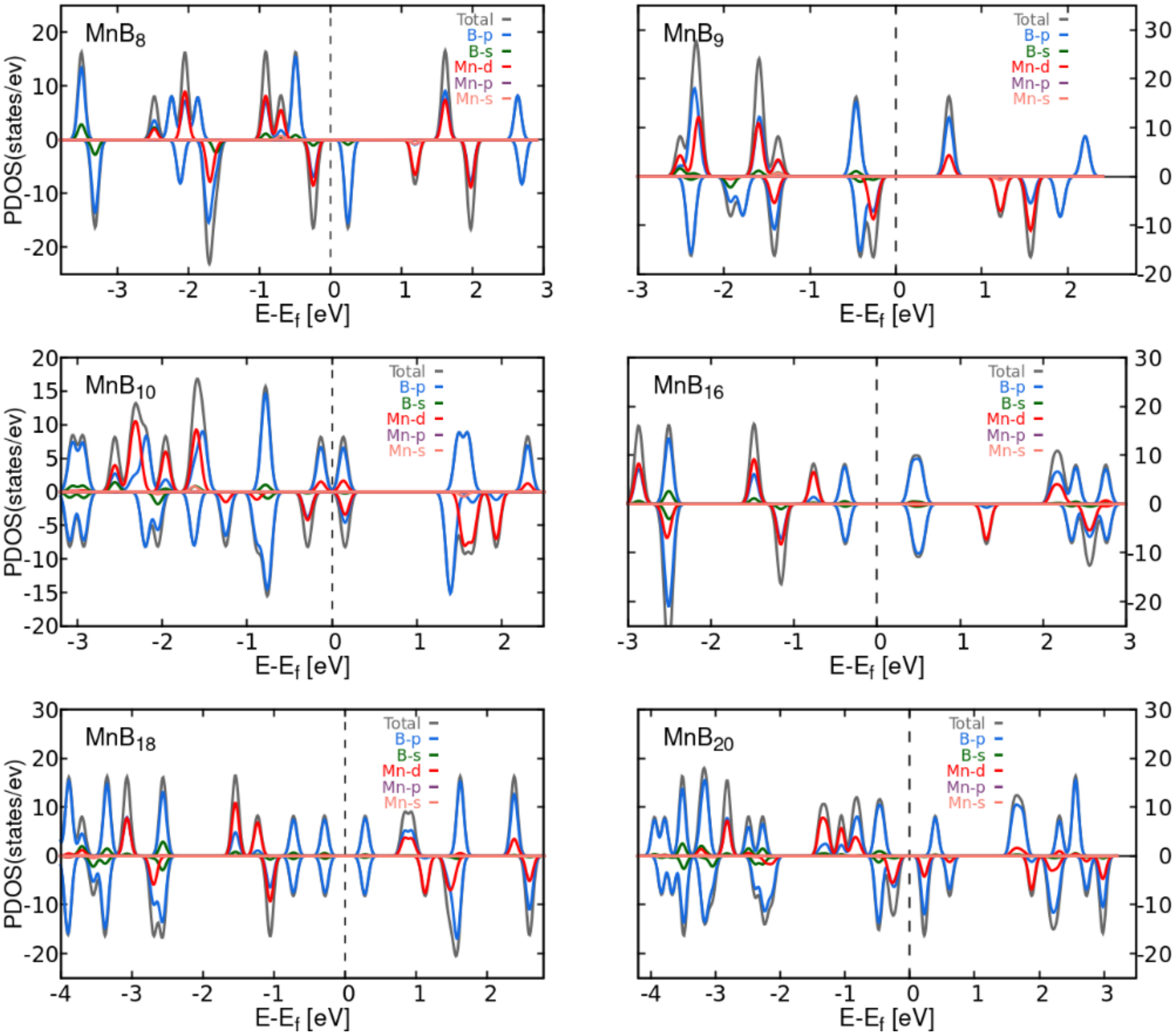

Figure S3. The PDOS for Mn-doped ring and drum structures.

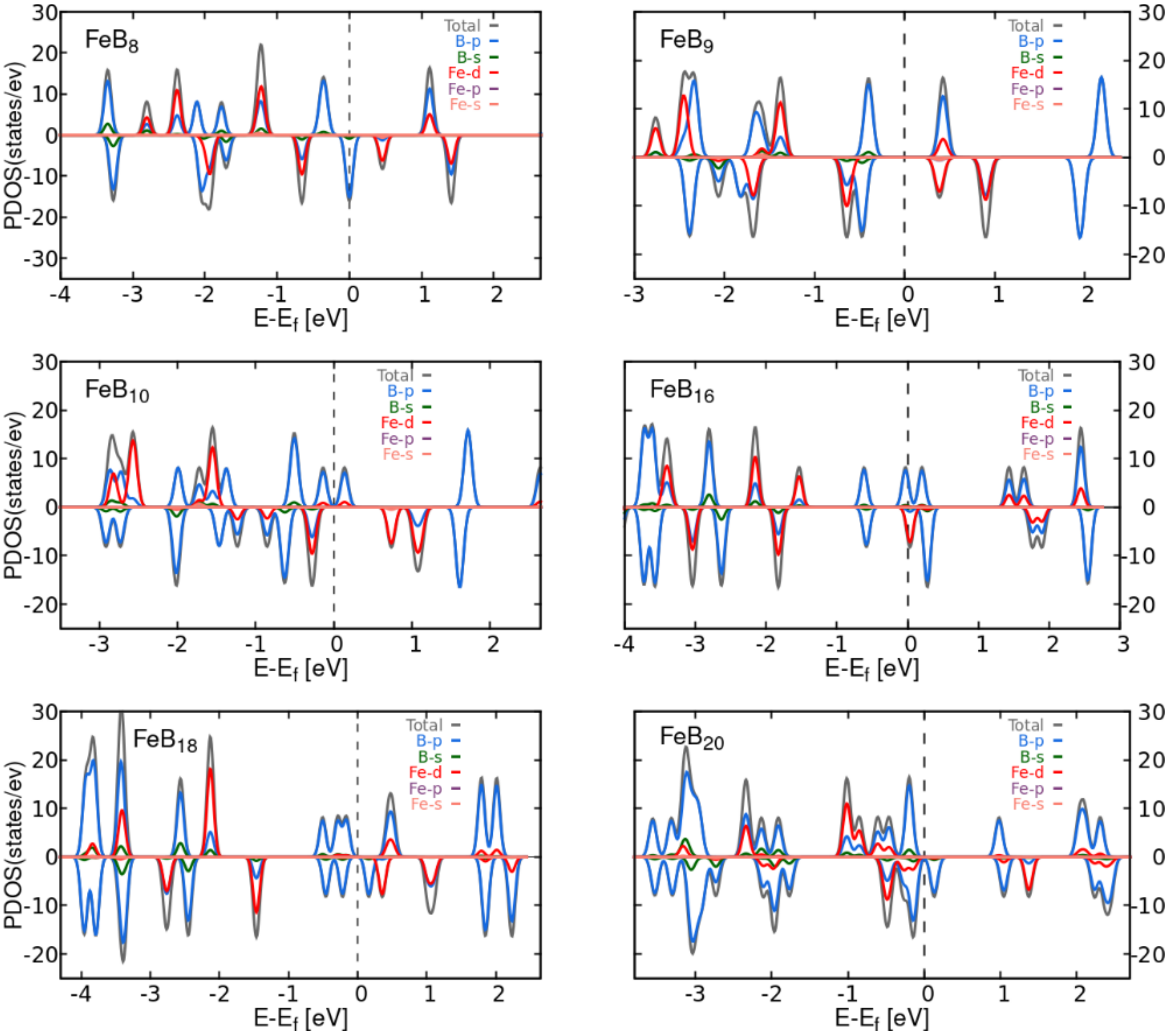

Figure S4. The PDOS for Fe-doped ring and drum structures.

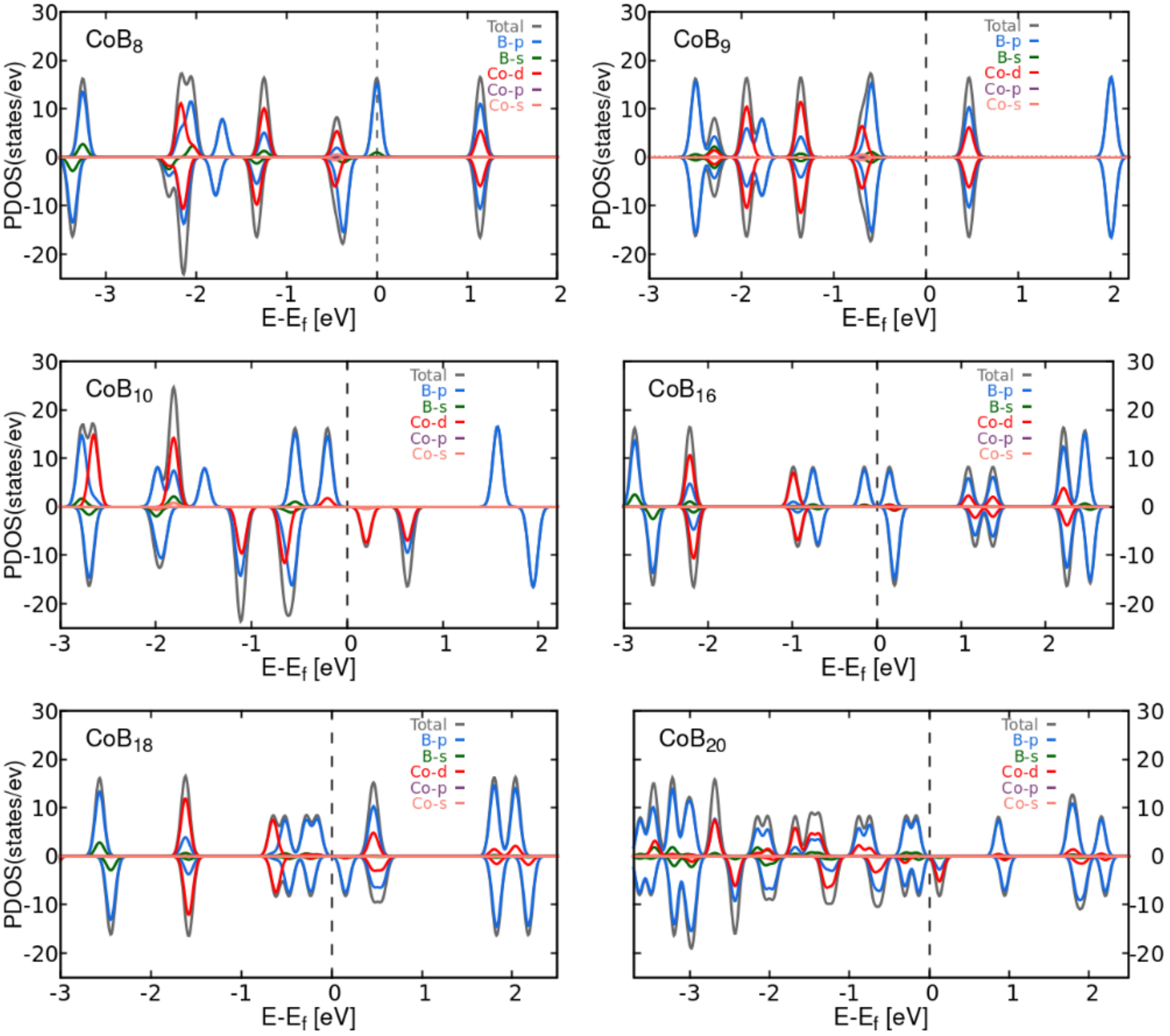

Figure S5. The PDOS for Co-doped ring and drum structures.

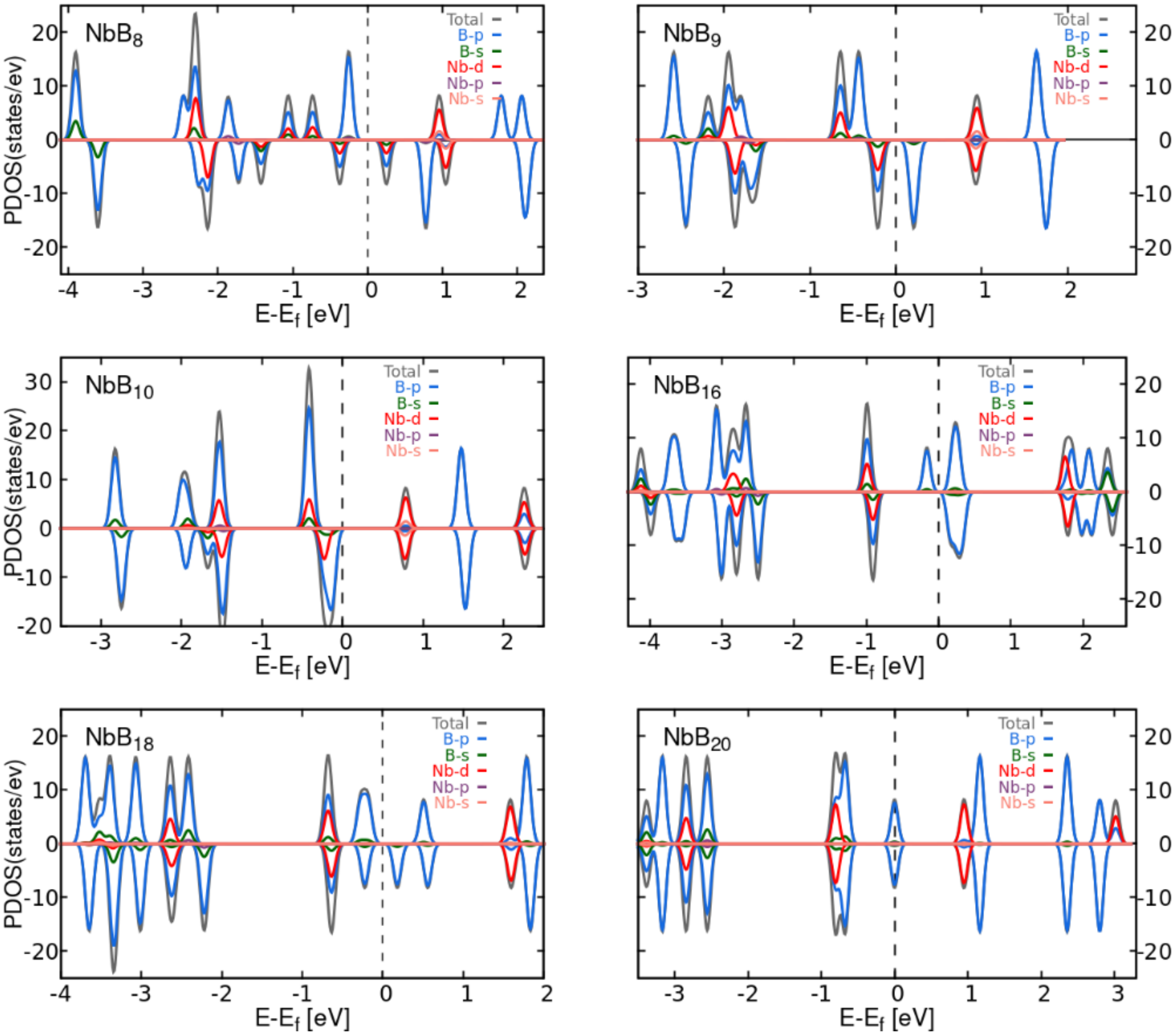

Figure S6. The PDOS for Nb-doped ring and drum structures.

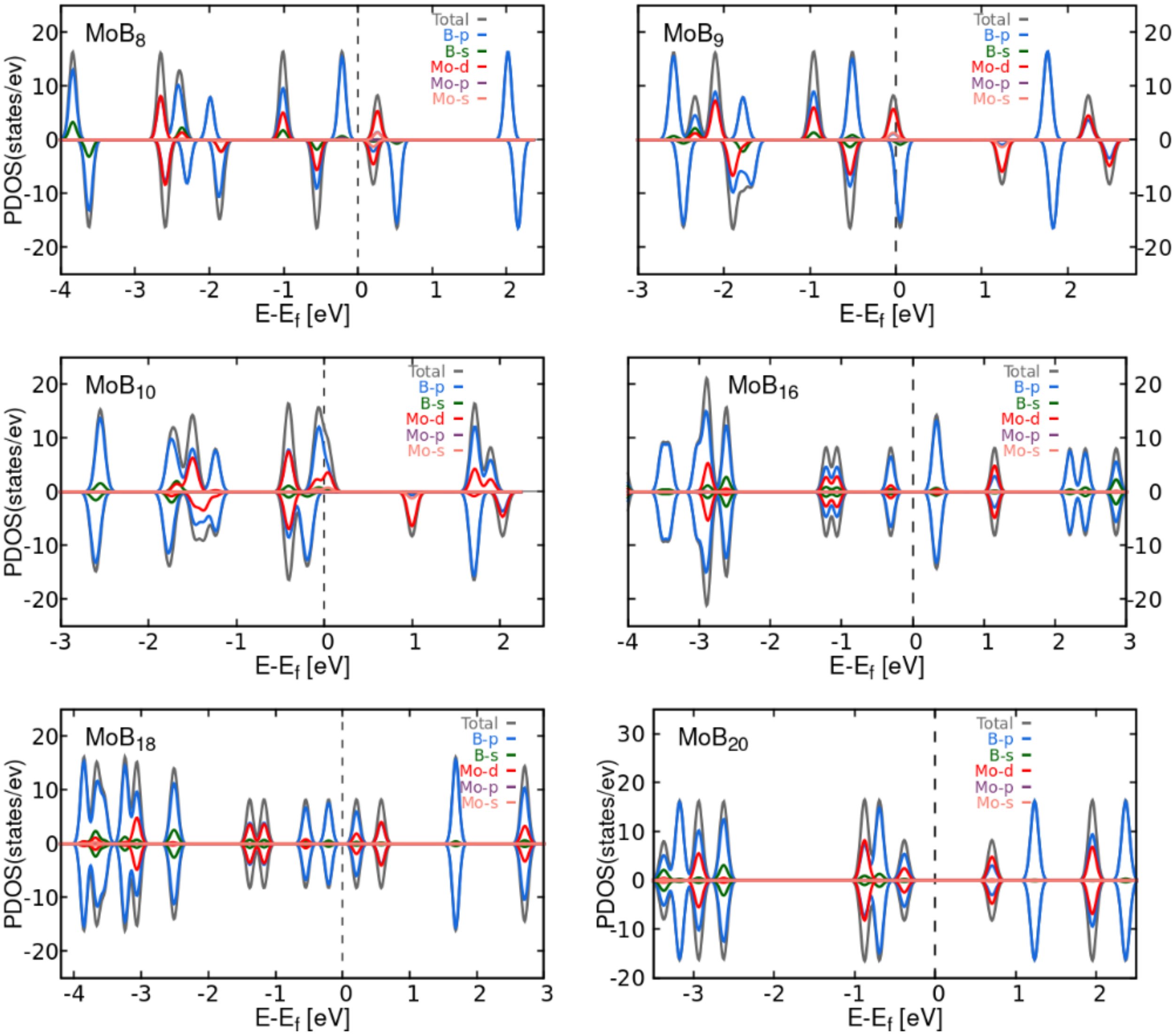

Figure S7. The PDOS for Mo-doped ring and drum structures.

SII. PHONON FREQUENCIES FOR EACH CLUSTER

The phonon frequencies for all the studied clusters are given in Tabs. from S1 to S14. The clusters point group symmetries specified in each table are given as identified by the Quantum ESPRESSO code.

Table S1: Phonon frequencies of Ti-doped ring configurations. Infrared (I) and Raman (R) active modes are indicated.

Frequency	TiB_8 (cm^{-1})	C_s	TiB_9 (cm^{-1})	C_1	TiB_{10} (cm^{-1})	C_1
f_1	206.22	A' I+R	123.91	A I+R	85.00	A I+R
f_2	224.69	A' I+R	185.43	A I+R	89.75	A I+R
f_3	231.04	A" I+R	188.06	A I+R	116.18	A I+R
f_4	370.44	A' I+R	261.34	A I+R	224.62	A I+R
f_5	373.23	A" I+R	268.23	A I+R	238.38	A I+R
f_6	379.34	A' I+R	328.19	A I+R	245.26	A I+R
f_7	380.46	A" I+R	331.54	A I+R	248.23	A I+R
f_8	384.39	A' I+R	365.58	A I+R	265.62	A I+R
f_9	402.30	A" I+R	366.66	A I+R	273.03	A I+R
f_{10}	421.33	A' I+R	448.66	A I+R	331.53	A I+R
f_{11}	513.91	A' I+R	449.73	A I+R	340.16	A I+R
f_{12}	515.43	A" I+R	463.66	A I+R	421.58	A I+R
f_{13}	554.40	A' I+R	464.76	A I+R	427.34	A I+R
f_{14}	685.14	A' I+R	531.73	A I+R	449.42	A I+R
f_{15}	802.41	A" I+R	539.14	A I+R	460.10	A I+R
f_{16}	804.23	A' I+R	644.94	A I+R	468.42	A I+R
f_{17}	1085.07	A' I+R	756.93	A I+R	487.28	A I+R
f_{18}	1086.95	A" I+R	781.64	A I+R	587.46	A I+R
f_{19}	1307.26	A' I+R	943.35	A I+R	722.89	A I+R
f_{20}	1307.79	A" I+R	951.86	A I+R	730.98	A I+R
f_{21}	1424.21	A" I+R	1243.03	A I+R	1038.90	A I+R
f_{22}			1248.48	A I+R	1041.70	A I+R

f_{23}			1495.99	A I+R	1228.73	A I+R
f_{24}			1496.94	A I+R	1230.83	A I+R
f_{25}					1552.41	A I+R
f_{26}					1555.63	A I+R
f_{27}					1640.06	A I+R

Table S2: Phonon frequencies of Ti-doped drum configurations. Infrared (I) and Raman (R) active modes are indicated.

Frequency	TiB_{16} (cm^{-1})	C_s	TiB_{18} (cm^{-1})	C_1	TiB_{20} (cm^{-1})	C_1
f_1	48.41		65.62	A I+R	78.17	A I+R
f_2	71.92		180.53	A I+R	88.59	A I+R
f_3	213.37		187.50	A I+R	124.49	A I+R
f_4	216.99		207.90	A I+R	138.76	A I+R
f_5	230.46		244.07	A I+R	154.57	A I+R
f_6	268.49		271.62	A I+R	213.64	A I+R
f_7	335.67		303.26	A I+R	220.79	A I+R
f_8	363.85		323.22	A I+R	222.30	A I+R
f_9	377.72		357.73	A I+R	231.28	A I+R
f_{10}	381.98		375.13	A I+R	287.81	A I+R
f_{11}	391.88		396.60	A I+R	291.07	A I+R
f_{12}	409.97		406.65	A I+R	341.48	A I+R
f_{13}	411.31		414.40	A I+R	345.88	A I+R
f_{14}	428.03	A” I+R	427.55	A I+R	357.11	A I+R
f_{15}	461.74		431.97	A I+R	372.06	A I+R
f_{16}	514.61		446.47	A I+R	375.65	A I+R
f_{17}	516.26		448.86	A I+R	399.73	A I+R
f_{18}	554.13		451.60	A I+R	405.12	A I+R
f_{19}	557.32		465.22	A I+R	414.74	A I+R
f_{20}	561.38		510.76	A I+R	418.21	A I+R

f_{21}	572.79		521.72	A I+R	432.87	A I+R
f_{22}	586.42		530.90	A I+R	469.65	A I+R
f_{23}	594.42		542.26	A I+R	473.51	A I+R
f_{24}	601.75		548.19	A I+R	480.53	A I+R
f_{25}	605.63		575.29	A I+R	487.47	A I+R
f_{26}	629.18	A" I+R	579.61	A I+R	490.59	A I+R
f_{27}	653.86	A" I+R	582.88	A I+R	496.64	A I+R
f_{28}	663.65	A' I+R	589.95	A I+R	498.63	A I+R
f_{29}	671.49	A" I+R	607.69	A I+R	504.49	A I+R
f_{30}	702.62	A' I+R	623.93	A I+R	532.31	A I+R
f_{31}	780.41		639.71	A I+R	539.53	A I+R
f_{32}	801.22		644.62	A I+R	548.41	A I+R
f_{33}	801.60		655.34	A I+R	552.87	A I+R
f_{34}	849.86		663.95	A I+R	576.70	A I+R
f_{35}	851.32		743.02	A I+R	584.73	A I+R
f_{36}	974.28		748.57	A I+R	596.63	A I+R
f_{37}	982.95		760.10	A I+R	598.03	A I+R
f_{38}	1028.34	A' I+R	826.29	A I+R	621.06	A I+R
f_{39}	1028.95	A" I+R	838.67	A I+R	690.61	A I+R
f_{40}	1078.20	A' I+R	949.52	A I+R	698.59	A I+R
f_{41}	1084.24	A" I+R	991.57	A I+R	700.29	A I+R
f_{42}	1161.83	A' I+R	1028.22	A I+R	809.58	A I+R
f_{43}	1162.75	A" I+R	1047.53	A I+R	811.25	A I+R
f_{44}	1170.47		1088.96	A I+R	963.38	A I+R
f_{45}	1172.85		1163.06	A I+R	966.50	A I+R
f_{46}			1174.51	A I+R	1049.24	A I+R
f_{47}			1186.85	A I+R	1050.51	A I+R
f_{48}			1205.38	A I+R	1211.38	A I+R
f_{49}			1230.20	A I+R	1214.89	A I+R
f_{50}			1232.55	A I+R	1223.34	A I+R

f_{51}			1246.96	A I+R	1226.35	A I+R
f_{52}					1326.76	A I+R
f_{53}					1330.67	A I+R
f_{54}					1350.50	A I+R
f_{55}					1352.86	A I+R
f_{56}					1361.82	A I+R
f_{57}					1364.64	A I+R

Table S3: Phonon frequencies for Cr-doped ring configurations. Infrared (I) and Raman (R) active modes are indicated.

Frequency	CrB_8 (cm^{-1})	C_s	CrB_9 (cm^{-1})	C_1	CrB_{10} (cm^{-1})	C_1
f_1	87.93		196.47	A I+R	44.03	A I+R
f_2	89.11		276.39	A I+R	74.20	A I+R
f_3	248.46	A" I+R	279.87	A I+R	107.17	A I+R
f_4	264.32	A' I+R	302.40	A I+R	145.49	A I+R
f_5	396.19		356.02	A I+R	163.91	A I+R
f_6	400.86		364.95	A I+R	188.54	A I+R
f_7	403.44		397.43	A I+R	207.17	A I+R
f_8	436.04	A" I+R	406.44	A I+R	218.45	A I+R
f_9	439.34	A' I+R	414.43	A I+R	229.01	A I+R
f_{10}	488.31	A' I+R	474.42	A I+R	232.47	A I+R
f_{11}	539.88		484.04	A I+R	279.80	A I+R
f_{12}	550.93		514.73	A I+R	326.56	A I+R
f_{13}	553.45		527.60	A I+R	377.09	A I+R
f_{14}	582.26	A' I+R	605.38	A I+R	389.50	A I+R
f_{15}	732.59	A' I+R	647.37	A I+R	391.50	A I+R
f_{16}	834.20		718.64	A I+R	405.31	A I+R
f_{17}	841.30		784.06	A I+R	443.08	A I+R
f_{18}	1058.24	A" I+R	828.17	A I+R	574.83	A I+R

f_{19}	1070.54	A' I+R	1042.96	A I+R	738.00	A I+R
f_{20}	1185.26		1060.20	A I+R	745.55	A I+R
f_{21}	1187.53		1075.27	A I+R	1058.42	A I+R
f_{22}			1077.68	A I+R	1072.13	A I+R
f_{23}			1526.55	A I+R	1116.87	A I+R
f_{24}			1538.10	A I+R	1334.90	A I+R
f_{25}					1375.72	A I+R
f_{26}					1612.60	A I+R
f_{27}					1704.65	A I+R

Table S4: Phonon frequencies for Cr-doped drum configurations. Infrared (I) and Raman (R) active modes are indicated.

Frequency	CrB_{16} (cm^{-1})	C_s	CrB_{18} (cm^{-1})	C_1	CrB_{20} (cm^{-1})	C_1
f_1	160.53	A" I+R	59.27	A I+R	89.91	A I+R
f_2	200.05	A' I+R	151.83	A I+R	154.53	A I+R
f_3	281.70	A' I+R	215.21	A I+R	154.66	A I+R
f_4	317.92	A" I+R	226.49	A I+R	158.42	A I+R
f_5	344.98	A' I+R	233.87	A I+R	174.50	A I+R
f_6	365.20	A" I+R	299.42	A I+R	259.01	A I+R
f_7	368.09	A' I+R	302.44	A I+R	275.03	A I+R
f_8	393.15	A" I+R	325.75	A I+R	287.62	A I+R
f_9	399.64	A' I+R	328.19	A I+R	302.35	A I+R
f_{10}	413.38	A' I+R	344.05	A I+R	321.39	A I+R
f_{11}	428.04	A" I+R	345.22	A I+R	341.26	A I+R
f_{12}	437.21	A' I+R	373.51	A I+R	366.58	A I+R
f_{13}	447.12	A" I+R	380.91	A I+R	371.95	A I+R
f_{14}	493.68	A" I+R	388.30	A I+R	386.25	A I+R
f_{15}	497.23	A' I+R	391.79	A I+R	389.54	A I+R

f_{16}	500.33	A" I+R	423.15	A I+R	409.16	A I+R
f_{17}	502.45	A' I+R	436.78	A I+R	432.05	A I+R
f_{18}	541.59	A" I+R	438.11	A I+R	444.83	A I+R
f_{19}	550.71	A" I+R	517.63	A I+R	461.68	A I+R
f_{20}	559.29	A' I+R	524.46	A I+R	468.89	A I+R
f_{21}	569.90	A' I+R	527.23	A I+R	483.19	A I+R
f_{22}	586.55	A' I+R	532.49	A I+R	490.07	A I+R
f_{23}	605.05	A" I+R	550.12	A I+R	509.20	A I+R
f_{24}	611.36	A' I+R	556.48	A I+R	524.29	A I+R
f_{25}	614.00	A' I+R	563.54	A I+R	530.43	A I+R
f_{26}	625.91	A" I+R	564.14	A I+R	534.86	A I+R
f_{27}	640.47	A' I+R	571.50	A I+R	554.07	A I+R
f_{28}	641.12	A" I+R	574.36	A I+R	586.46	A I+R
f_{29}	654.37	A" I+R	581.64	A I+R	591.43	A I+R
f_{30}	670.02	A' I+R	605.72	A I+R	610.68	A I+R
f_{31}	725.02	A' I+R	612.56	A I+R	623.68	A I+R
f_{32}	797.45	A' I+R	620.72	A I+R	656.04	A I+R
f_{33}	798.26	A" I+R	626.94	A I+R	661.97	A I+R
f_{34}	886.53	A' I+R	634.23	A I+R	664.99	A I+R
f_{35}	893.82	A" I+R	713.16	A I+R	671.96	A I+R
f_{36}	1021.65	A" I+R	751.79	A I+R	676.42	A I+R
f_{37}	1040.64	A' I+R	754.42	A I+R	712.89	A I+R
f_{38}	1109.57	A" I+R	853.21	A I+R	717.67	A I+R
f_{39}	1121.10	A' I+R	856.63	A I+R	729.46	A I+R
f_{40}	1164.61	A' I+R	1014.90	A I+R	751.05	A I+R
f_{41}	1173.34	A" I+R	1018.72	A I+R	803.93	A I+R
f_{42}	1227.11	A' I+R	1079.29	A I+R	811.58	A I+R
f_{43}	1227.70	A' I+R	1088.07	A I+R	834.20	A I+R
f_{44}	1269.63	A" I+R	1222.28	A I+R	934.76	A I+R
f_{45}	1274.26	A" I+R	1225.04	A I+R	947.80	A I+R

f_{46}			1232.58	A I+R	1011.78	A I+R
f_{47}			1256.32	A I+R	1014.47	A I+R
f_{48}			1256.77	A I+R	1132.64	A I+R
f_{49}			1262.48	A I+R	1139.91	A I+R
f_{50}			1312.27	A I+R	1159.33	A I+R
f_{51}			1314.97	A I+R	1167.21	A I+R
f_{52}					1224.01	A I+R
f_{53}					1226.23	A I+R
f_{54}					1229.13	A I+R
f_{55}					1262.29	A I+R
f_{56}					1285.89	A I+R
f_{57}					1309.64	A I+R

Table S5: Phonon frequencies of Mn-doped ring configurations. Infrared (I) and Raman (R) active modes are indicated.

Frequency	MnB_8 (cm^{-1})	C_s	MnB_9 (cm^{-1})	C_1	MnB_{10} (cm^{-1})	C_1
f_1	140.21		135.68	A I+R	69.60	A I+R
f_2	250.98	A' I+R	157.36	A I+R	101.24	A I+R
f_3	267.65	A" I+R	160.88	A I+R	119.25	A I+R
f_4	411.25		270.87	A I+R	141.23	A I+R
f_5	421.56		284.32	A I+R	145.87	A I+R
f_6	431.86		299.87	A I+R	171.55	A I+R
f_7	433.27		306.29	A I+R	200.51	A I+R
f_8	445.64		338.21	A I+R	239.89	A I+R
f_9	455.34		339.12	A I+R	247.45	A I+R
f_{10}	457.91		366.86	A I+R	260.50	A I+R
f_{11}	472.37	A' I+R	383.65	A I+R	320.72	A I+R
f_{12}	490.44		441.44	A I+R	375.16	A I+R
f_{13}	526.06		447.02	A I+R	386.26	A I+R

f_{14}	745.05		454.49	A I+R	404.56	A I+R
f_{15}	844.63		454.85	A I+R	421.73	A I+R
f_{16}	844.85		613.90	A I+R	425.31	A I+R
f_{17}	1030.34		784.11	A I+R	454.09	A I+R
f_{18}	1034.55		787.82	A I+R	563.31	A I+R
f_{19}	1121.44		1088.84	A I+R	709.87	A I+R
f_{20}	1122.43		1094.68	A I+R	761.80	A I+R
f_{21}	1455.81	A" I+R	1358.7	A I+R	1011.35	A I+R
f_{22}			1360.87	A I+R	1058.85	A I+R
f_{23}			1549.66	A I+R	1254.93	A I+R
f_{24}			1661.71	A I+R	1312.99	A I+R
f_{25}					1342.30	A I+R
f_{26}					1587.27	A I+R
f_{27}					1677.08	A I+R

Table S6: Phonon frequencies of Mn-doped drum configurations. Infrared (I) and Raman (R) active modes are indicated.

Frequency	MnB_{16} (cm^{-1})	C_s	MnB_{18} (cm^{-1})	C_1	MnB_{20} (cm^{-1})	C_1
f_1	122.13	A" I+R	130.70	A I+R	27.00	A I+R
f_2	148.07	A' I+R	205.58	A I+R	36.22	A I+R
f_3	259.72	A' I+R	213.60	A I+R	82.05	A I+R
f_4	295.09	A" I+R	241.10	A I+R	152.93	A I+R
f_5	301.85	A" I+R	247.32	A I+R	154.10	A I+R
f_6	313.14	A' I+R	282.31	A I+R	249.05	A I+R
f_7	333.82	A" I+R	285.10	A I+R	268.34	A I+R
f_8	344.93	A' I+R	328.76	A I+R	305.02	A I+R
f_9	374.41	A' I+R	340.91	A I+R	319.88	A I+R
f_{10}	386.44	A" I+R	348.24	A I+R	324.53	A I+R

f_{11}	402.75	A' I+R	354.95	A I+R	337.40	A I+R
f_{12}	435.67	A" I+R	362.90	A I+R	347.36	A I+R
f_{13}	439.86	A' I+R	401.81	A I+R	364.77	A I+R
f_{14}	471.63	A" I+R	411.11	A I+R	385.24	A I+R
f_{15}	491.09	A" I+R	415.42	A I+R	391.83	A I+R
f_{16}	503.18	A' I+R	451.19	A I+R	397.88	A I+R
f_{17}	508.90	A" I+R	465.55	A I+R	407.75	A I+R
f_{18}	509.81	A' I+R	466.85	A I+R	421.61	A I+R
f_{19}	512.64	A' I+R	510.58	A I+R	426.66	A I+R
f_{20}	529.24	A" I+R	514.66	A I+R	444.57	A I+R
f_{21}	563.75	A' I+R	515.79	A I+R	470.51	A I+R
f_{22}	567.12	A" I+R	528.36	A I+R	499.25	A I+R
f_{23}	579.15	A' I+R	537.72	A I+R	512.44	A I+R
f_{24}	595.89	A' I+R	554.22	A I+R	531.89	A I+R
f_{25}	599.41	A" I+R	559.33	A I+R	542.92	A I+R
f_{26}	600.40	A' I+R	568.67	A I+R	551.45	A I+R
f_{27}	627.28	A" I+R	577.64	A I+R	557.88	A I+R
f_{28}	627.91	A' I+R	590.77	A I+R	566.20	A I+R
f_{29}	648.99	A" I+R	598.35	A I+R	582.03	A I+R
f_{30}	661.58	A' I+R	609.19	A I+R	592.69	A I+R
f_{31}	712.79	A' I+R	614.58	A I+R	595.63	A I+R
f_{32}	797.47	A' I+R	624.25	A I+R	626.58	A I+R
f_{33}	799.21	A" I+R	635.05	A I+R	638.04	A I+R
f_{34}	898.04	A' I+R	636.61	A I+R	651.69	A I+R
f_{35}	899.22	A" I+R	705.18	A I+R	660.06	A I+R
f_{36}	1037.5	A" I+R	745.29	A I+R	672.36	A I+R
f_{37}	1052.52	A' I+R	747.39	A I+R	710.48	A I+R
f_{38}	1123.48	A" I+R	848.99	A I+R	716.24	A I+R
f_{39}	1136.15	A' I+R	851.68	A I+R	722.12	A I+R
f_{40}	1196.01	A' I+R	996.60	A I+R	738.47	A I+R

f_{41}	1205.92	A" I+R	1040.81	A I+R	798.34	A I+R
f_{42}	1244.24	A' I+R	1070.53	A I+R	816.20	A I+R
f_{43}	1270.74	A" I+R	1089.35	A I+R	833.16	A I+R
f_{44}	1277.74	A' I+R	1248.26	A I+R	945.39	A I+R
f_{45}	1283.42	A" I+R	1249.55	A I+R	947.22	A I+R
f_{46}			1256.63	A I+R	1000.55	A I+R
f_{47}			1257.30	A I+R	1001.71	A I+R
f_{48}			1277.30	A I+R	1159.98	A I+R
f_{49}			1323.45	A I+R	1163.05	A I+R
f_{50}			1327.24	A I+R	1176.67	A I+R
f_{51}			1340.70	A I+R	1176.92	A I+R
f_{52}					1235.72	A I+R
f_{53}					1239.22	A I+R
f_{54}					1244.10	A I+R
f_{55}					1247.03	A I+R
f_{56}					1293.39	A I+R
f_{57}					1297.31	A I+R

Table S7: Phonon frequencies of Fe-doped ring configurations. Infrared (I) and Raman (R) active modes are indicated.

Frequency	FeB_8 (cm^{-1})	C_1	FeB_9 (cm^{-1})	C_1	FeB_{10} (cm^{-1})	C_1
f_1	133.40	A I+R	128.48	A I+R	-22.15	A I+R
f_2	224.32	A I+R	134.08	A I+R	53.03	A I+R
f_3	226.41	A I+R	145.47	A I+R	62.54	A I+R
f_4	383.79	A I+R	267.53	A I+R	121.02	A I+R
f_5	393.28	A I+R	276.13	A I+R	147.13	A I+R
f_6	405.13	A I+R	302.00	A I+R	178.08	A I+R
f_7	409.61	A I+R	303.36	A I+R	195.37	A I+R
f_8	418.95	A I+R	314.57	A I+R	197.58	A I+R

f_9	420.62	A I+R	324.97	A I+R	214.59	A I+R
f_{10}	467.53	A I+R	346.02	A I+R	255.09	A I+R
f_{11}	476.08	A I+R	353.79	A I+R	277.24	A I+R
f_{12}	477.54	A I+R	396.97	A I+R	328.88	A I+R
f_{13}	520.92	A I+R	404.54	A I+R	354.13	A I+R
f_{14}	712.11	A I+R	452.36	A I+R	363.65	A I+R
f_{15}	840.35	A I+R	453.69	A I+R	368.98	A I+R
f_{16}	843.51	A I+R	639.43	A I+R	402.22	A I+R
f_{17}	1095.31	A I+R	796.92	A I+R	435.17	A I+R
f_{18}	1103.15	A I+R	805.37	A I+R	576.90	A I+R
f_{19}	1331.41	A I+R	1134.73	A I+R	722.45	A I+R
f_{20}	1343.31	A I+R	1140.10	A I+R	782.20	A I+R
f_{21}	1465.65	A I+R	1435.83	A I+R	1027.66	A I+R
f_{22}			1437.46	A I+R	1084.49	A I+R
f_{23}			1618.87	A I+R	1373.17	A I+R
f_{24}			1620.88	A I+R	1380.96	A I+R
f_{25}					1614.33	A I+R
f_{26}					1626.26	A I+R
f_{27}					1714.97	A I+R

Table S8: Phonon frequencies of Fe-doped drum configurations. Infrared (I) and Raman (R) active modes are indicated.

frequency	FeB_{16} (cm^{-1})	C_s	FeB_{18} (cm^{-1})	C_1	FeB_{20} (cm^{-1})	C_1
f_1	75.14		79.27	A I+R	64.30	A I+R
f_2	121.32		116.64	A I+R	78.44	A I+R
f_3	129.29		156.43	A I+R	144.22	A I+R
f_4	141.11	A” I+R	196.52	A I+R	146.62	A I+R
f_5	263.02		222.01	A I+R	176.12	A I+R
f_6	282.42		236.64	A I+R	193.43	A I+R

f_7	329.72		250.30	A I+R	258.13	A I+R
f_8	341.01		308.08	A I+R	294.80	A I+R
f_9	352.32		309.17	A I+R	301.95	A I+R
f_{10}	357.58		318.45	A I+R	319.23	A I+R
f_{11}	358.23		326.52	A I+R	324.93	A I+R
f_{12}	397.74		327.83	A I+R	343.10	A I+R
f_{13}	401.93		377.66	A I+R	360.90	A I+R
f_{14}	423.08		396.03	A I+R	370.68	A I+R
f_{15}	427.67	A" I+R	398.02	A I+R	381.40	A I+R
f_{16}	445.85	A' I+R	443.40	A I+R	401.29	A I+R
f_{17}	496.75		453.10	A I+R	415.90	A I+R
f_{18}	504.06		459.54	A I+R	422.69	A I+R
f_{19}	507.18		473.23	A I+R	435.98	A I+R
f_{20}	518.93		477.45	A I+R	458.96	A I+R
f_{21}	529.96		479.29	A I+R	493.18	A I+R
f_{22}	530.70		501.22	A I+R	497.50	A I+R
f_{23}	537.57		523.79	A I+R	525.10	A I+R
f_{24}	543.55		526.24	A I+R	558.65	A I+R
f_{25}	558.71		528.54	A I+R	565.16	A I+R
f_{26}	567.45		544.09	A I+R	580.26	A I+R
f_{27}	572.90	A' I+R	549.00	A I+R	616.03	A I+R
f_{28}	577.23	A" I+R	554.77	A I+R	622.21	A I+R
f_{29}	614.54		559.19	A I+R	628.83	A I+R
f_{30}	649.37	A' I+R	563.51	A I+R	637.04	A I+R
f_{31}	685.97		575.72	A I+R	651.22	A I+R
f_{32}	798.02		585.47	A I+R	669.40	A I+R
f_{33}	800.19		587.52	A I+R	677.94	A I+R
f_{34}	824.08	A" I+R	609.16	A I+R	690.66	A I+R
f_{35}	889.22		658.86	A I+R	693.92	A I+R
f_{36}	890.37		745.59	A I+R	712.02	A I+R

f_{37}	1033.99	A' I+R	751.48	A I+R	720.57	A I+R
f_{38}	1073.42	A" I+R	847.73	A I+R	726.85	A I+R
f_{39}	1101.09	A' I+R	850.24	A I+R	779.83	A I+R
f_{40}	1112.60	A' I+R	1014.30	A I+R	790.20	A I+R
f_{41}	1141.75	A" I+R	1035.94	A I+R	819.42	A I+R
f_{42}	1223.30	A" I+R	1068.58	A I+R	850.42	A I+R
f_{43}	1223.32	A' I+R	1076.31	A I+R	857.09	A I+R
f_{44}	1270.65		1245.54	A I+R	927.42	A I+R
f_{45}	1272.06		1261.98	A I+R	958.75	A I+R
f_{46}			1264.37	A I+R	998.25	A I+R
f_{47}			1270.61	A I+R	1014.79	A I+R
f_{48}			1271.87	A I+R	1026.92	A I+R
f_{49}			1331.34	A I+R	1121.23	A I+R
f_{50}			1331.72	A I+R	1122.90	A I+R
f_{51}			1355.65	A I+R	1154.21	A I+R
f_{52}					1163.22	A I+R
f_{53}					1213.52	A I+R
f_{54}					1216.40	A I+R
f_{55}					1225.36	A I+R
f_{56}					1249.61	A I+R
f_{57}					1264.58	A I+R

Table S9: Phonon frequencies of Co-doped ring configurations. Infrared (I) and Raman (R) active modes are indicated.

Frequency	CoB_8 (cm^{-1})	C_1	CoB_9 (cm^{-1})	C_1	CoB_{10} (cm^{-1})	C_1
f_1	132.22	A I+R	102.07	A I+R	78.05	A I+R
f_2	226.44	A I+R	108.75	A I+R	95.65	A I+R
f_3	227.15	A I+R	174.92	A I+R	105.29	A I+R
f_4	387.23	A I+R	270.40	A I+R	114.93	A I+R

f_5	394.42	A I+R	272.27	A I+R	123.21	A I+R
f_6	418.58	A I+R	286.67	A I+R	130.86	A I+R
f_7	419.63	A I+R	297.14	A I+R	178.84	A I+R
f_8	420.75	A I+R	301.72	A I+R	200.91	A I+R
f_9	427.31	A I+R	304.33	A I+R	213.54	A I+R
f_{10}	475.59	A I+R	329.26	A I+R	217.34	A I+R
f_{11}	476.76	A I+R	337.61	A I+R	251.07	A I+R
f_{12}	485.87	A I+R	379.35	A I+R	323.01	A I+R
f_{13}	531.35	A I+R	387.30	A I+R	331.76	A I+R
f_{14}	738.23	A I+R	438.29	A I+R	359.37	A I+R
f_{15}	846.01	A I+R	439.58	A I+R	375.51	A I+R
f_{16}	847.85	A I+R	651.98	A I+R	398.13	A I+R
f_{17}	1101.23	A I+R	810.22	A I+R	437.11	A I+R
f_{18}	1103.46	A I+R	811.89	A I+R	560.52	A I+R
f_{19}	1353.44	A I+R	1150.56	A I+R	747.49	A I+R
f_{20}	1358.81	A I+R	1157.41	A I+R	748.06	A I+R
f_{21}	1479.23	A I+R	1454.00	A I+R	1075.52	A I+R
f_{22}			1457.51	A I+R	1077.22	A I+R
f_{23}			1646.17	A I+R	1367.16	A I+R
f_{24}			1648.97	A I+R	1368.35	A I+R
f_{25}					1618.81	A I+R
f_{26}					1622.29	A I+R
f_{27}					1725.10	A I+R

Table S10: Phonon frequencies of Co-doped drum configurations. Infrared (I) and Raman (R) active modes are indicated.

Frequency	CoB_{16} (cm^{-1})	C_s	CoB_{18} (cm^{-1})	C_1	CoB_{20} (cm^{-1})	C_1
f_1	55.24		70.30	A I+R	90.34	A I+R
f_2	125.56	A" I+R	104.90	A I+R	130.45	A I+R
f_3	183.41		186.93	A I+R	144.81	A I+R
f_4	239.82		190.51	A I+R	154.66	A I+R
f_5	251.97		193.83	A I+R	173.13	A I+R
f_6	281.51		223.87	A I+R	214.68	A I+R
f_7	283.58		227.31	A I+R	293.36	A I+R
f_8	295.37		268.21	A I+R	303.80	A I+R
f_9	327.38		277.13	A I+R	315.26	A I+R
f_{10}	336.41		302.99	A I+R	330.09	A I+R
f_{11}	338.91		314.84	A I+R	345.59	A I+R
f_{12}	388.97		323.52	A I+R	363.58	A I+R
f_{13}	389.10		357.26	A I+R	369.65	A I+R
f_{14}	402.59		386.96	A I+R	382.94	A I+R
f_{15}	443.12	A' I+R	396.95	A I+R	389.96	A I+R
f_{16}	443.77	A" I+R	406.73	A I+R	412.65	A I+R
f_{17}	483.54		436.30	A I+R	420.45	A I+R
f_{18}	483.55		443.72	A I+R	442.17	A I+R
f_{19}	518.91		450.95	A I+R	448.84	A I+R
f_{20}	520.43		458.89	A I+R	482.05	A I+R
f_{21}	520.83		471.01	A I+R	508.91	A I+R
f_{22}	530.04	A' I+R	483.58	A I+R	511.74	A I+R
f_{23}	537.34	A" I+R	502.31	A I+R	546.64	A I+R
f_{24}	538.10	A' I+R	506.27	A I+R	552.08	A I+R
f_{25}	555.69	A" I+R	514.22	A I+R	581.40	A I+R
f_{26}	577.98		519.47	A I+R	583.95	A I+R

f_{27}	578.60		533.71	A I+R	600.39	A I+R
f_{28}	614.82		535.66	A I+R	636.88	A I+R
f_{29}	617.04		544.27	A I+R	645.18	A I+R
f_{30}	648.44	A' I+R	551.28	A I+R	663.60	A I+R
f_{31}	694.17		556.12	A I+R	667.42	A I+R
f_{32}	799.36		570.59	A I+R	678.98	A I+R
f_{33}	799.39		575.16	A I+R	693.57	A I+R
f_{34}	890.72		607.35	A I+R	698.88	A I+R
f_{35}	891.06		645.77	A I+R	705.84	A I+R
f_{36}	895.58		740.25	A I+R	725.20	A I+R
f_{37}	1034.15	A' I+R	744.15	A I+R	732.12	A I+R
f_{38}	1090.63	A" I+R	849.47	A I+R	742.46	A I+R
f_{39}	1119.24	A' I+R	854.15	A I+R	788.81	A I+R
f_{40}	1146.91	A" I+R	1020.57	A I+R	805.33	A I+R
f_{41}	1240.40		1042.72	A I+R	807.82	A I+R
f_{42}	1240.79		1071.67	A I+R	833.73	A I+R
f_{43}	1249.37	A' I+R	1080.23	A I+R	863.13	A I+R
f_{44}	1281.95		1263.77	A I+R	931.85	A I+R
f_{45}	1282.75		1265.38	A I+R	945.16	A I+R
f_{46}			1273.63	A I+R	997.06	A I+R
f_{47}			1279.82	A I+R	1005.73	A I+R
f_{48}			1280.97	A I+R	1119.18	A I+R
f_{49}			1342.26	A I+R	1128.13	A I+R
f_{50}			1347.29	A I+R	1156.08	A I+R
f_{51}			1364.44	A I+R	1159.55	A I+R
f_{52}					1192.41	A I+R
f_{53}					1198.65	A I+R
f_{54}					1223.64	A I+R
f_{55}					1227.45	A I+R
f_{56}					1252.21	A I+R

f_{57}					1257.99	A I+R

Table S11: Phonon frequencies of Nb-doped ring configurations. Infrared (I) and Raman (R) active modes are indicated.

Frequency	NbB_8 (cm^{-1})	C_{2v}	NbB_9 (cm^{-1})	C_s	NbB_{10} (cm^{-1})	C_1
f_1	45.69		93.35	A I+R	87.85	A I+R
f_2	313.89	A" I+R	231.47	A I+R	99.43	A I+R
f_3	319.69	A' I+R	239.29	A I+R	119.56	A I+R
f_4	385.82		324.59	A I+R	228.25	A I+R
f_5	391.04		326.83	A I+R	251.08	A I+R
f_6	480.65	A' I+R	399.08	A I+R	269.88	A I+R
f_7	482.80	A" I+R	403.12	A I+R	274.93	A I+R
f_8	490.75	A' I+R	422.12	A I+R	275.32	A I+R
f_9	524.98	A' I+R	427.38	A I+R	279.48	A I+R
f_{10}	566.78	A" I+R	478.30	A I+R	359.78	A I+R
f_{11}	605.52	A' I+R	485.41	A I+R	360.65	A I+R
f_{12}	616.09		501.33	A I+R	426.68	A I+R
f_{13}	620.13		502.90	A I+R	428.06	A I+R
f_{14}	723.57	A' I+R	504.64	A I+R	431.2	A I+R
f_{15}	766.64		513.64	A I+R	431.57	A I+R
f_{16}	766.89		671.58	A I+R	463.92	A I+R
f_{17}	880.82	A" I+R	754.23	A I+R	474.95	A I+R
f_{18}	896.65	A" I+R	755.89	A I+R	598.35	A I+R
f_{19}	911.61	A' I+R	933.68	A I+R	725.33	A I+R
f_{20}	1019.63	A" I+R	939.01	A I+R	741.39	A I+R
f_{21}	1024.44	A' I+R	1042.89	A I+R	1041.86	A I+R
f_{22}			1046.05	A I+R	1048.98	A I+R
f_{23}			1441.40	A I+R	1312.83	A I+R

f_{24}			1443.84	A I+R	1314.16	A I+R
f_{25}					1539.09	A I+R
f_{26}					1541.32	A I+R
f_{27}					1629.34	A I+R

Table S12: Phonon frequencies of Nb-doped drum configurations. Infrared (I) and Raman (R) active modes are indicated.

Frequency	NbB_{16} (cm^{-1})	C_s	NbB_{18} (cm^{-1})	C_1	NbB_{20} (cm^{-1})	C_1
f_1	153.38	A" I+R	38.60	A I+R	102.61	A I+R
f_2	225.89	A' I+R	107.72	A I+R	129.29	A I+R
f_3	255.94	A' I+R	177.78	A I+R	134.64	A I+R
f_4	371.24	A' I+R	284.27	A I+R	149.29	A I+R
f_5	381.25	A" I+R	302.70	A I+R	181.05	A I+R
f_6	390.64	A" I+R	308.98	A I+R	215.88	A I+R
f_7	395.53	A' I+R	316.19	A I+R	218.76	A I+R
f_8	402.44	A' I+R	326.75	A I+R	298.28	A I+R
f_9	411.50	A" I+R	331.19	A I+R	304.37	A I+R
f_{10}	435.82	A" I+R	392.56	A I+R	330.27	A I+R
f_{11}	437.24	A" I+R	399.84	A I+R	335.28	A I+R
f_{12}	452.89	A' I+R	407.55	A I+R	380.34	A I+R
f_{13}	463.47	A' I+R	410.92	A I+R	389.66	A I+R
f_{14}	475.30	A" I+R	419.69	A I+R	403.76	A I+R
f_{15}	477.29	A' I+R	444.91	A I+R	408.58	A I+R
f_{16}	508.80	A" I+R	450.70	A I+R	411.67	A I+R
f_{17}	513.09	A' I+R	453.16	A I+R	414.72	A I+R
f_{18}	526.48	A" I+R	455.63	A I+R	420.79	A I+R
f_{19}	529.77	A" I+R	505.88	A I+R	422.32	A I+R
f_{20}	548.57	A' I+R	513.86	A I+R	469.69	A I+R

f_{21}	585.34	A’ I+R	525.01	A I+R	473.67	A I+R
f_{22}	607.84	A” I+R	526.71	A I+R	488.11	A I+R
f_{23}	615.15	A’ I+R	530.15	A I+R	491.91	A I+R
f_{24}	620.61	A’ I+R	553.84	A I+R	498.13	A I+R
f_{25}	628.93	A” I+R	567.70	A I+R	502.23	A I+R
f_{26}	636.29	A’ I+R	568.91	A I+R	503.08	A I+R
f_{27}	651.43	A” I+R	585.49	A I+R	504.58	A I+R
f_{28}	675.07	A” I+R	585.71	A I+R	509.92	A I+R
f_{29}	678.25	A’ I+R	610.94	A I+R	518.68	A I+R
f_{30}	701.59	A’ I+R	616.56	A I+R	521.01	A I+R
f_{31}	736.31	A’ I+R	618.38	A I+R	529.44	A I+R
f_{32}	783.80	A” I+R	621.92	A I+R	533.12	A I+R
f_{33}	784.32	A’ I+R	623.28	A I+R	537.08	A I+R
f_{34}	806.59	A’ I+R	624.97	A I+R	553.72	A I+R
f_{35}	812.25	A” I+R	697.93	A I+R	555.91	A I+R
f_{36}	849.55	A” I+R	741.01	A I+R	569.10	A I+R
f_{37}	912.57	A” I+R	745.00	A I+R	570.58	A I+R
f_{38}	949.59	A’ I+R	825.52	A I+R	603.47	A I+R
f_{39}	965.49	A” I+R	826.19	A I+R	655.46	A I+R
f_{40}	983.48	A’ I+R	970.24	A I+R	702.13	A I+R
f_{41}	1013.57	A’ I+R	988.10	A I+R	705.11	A I+R
f_{42}	1037.14	A’ I+R	1023.17	A I+R	817.16	A I+R
f_{43}	1038.94	A” I+R	1043.28	A I+R	819.79	A I+R
f_{44}	1063.51	A’ I+R	1072.06	A I+R	976.61	A I+R
f_{45}	1093.04	A” I+R	1176.98	A I+R	980.59	A I+R
f_{46}			1178.71	A I+R	1051.48	A I+R
f_{47}			1196.05	A I+R	1053.57	A I+R
f_{48}			1197.44	A I+R	1242.33	A I+R
f_{49}			1224.51	A I+R	1242.87	A I+R
f_{50}			1226.53	A I+R	1253.91	A I+R

f_{51}			1260.09	A I+R	1255.97	A I+R
f_{52}					1363.29	A I+R
f_{53}					1365.27	A I+R
f_{54}					1372.29	A I+R
f_{55}					1374.83	A I+R
f_{56}					1389.72	A I+R
f_{57}					1390.28	A I+R

Table S13: Phonon frequencies of Mo-doped ring configurations. Infrared (I) and Raman (R) active modes are indicated.

Frequency	MoB_8 (cm^{-1})	C_{2v}	MoB_9 (cm^{-1})	C_s	MoB_{10} (cm^{-1})	C_1
f_1	36.02		96.98	A I+R	75.20	A I+R
f_2	283.32	A" I+R	190.06	A I+R	97.13	A I+R
f_3	295.36	A' I+R	190.86	A I+R	145.88	A I+R
f_4	359.78		317.12	A I+R	181.14	A I+R
f_5	369.94		324.29	A I+R	192.76	A I+R
f_6	467.31		379.84	A I+R	226.27	A I+R
f_7	471.56		380.44	A I+R	240.30	A I+R
f_8	515.87	A' I+R	387.91	A I+R	250.33	A I+R
f_9	518.61		396.99	A I+R	259.06	A I+R
f_{10}	532.09		467.66	A I+R	300.87	A I+R
f_{11}	549.84		470.01	A I+R	315.34	A I+R
f_{12}	563.40		485.96	A I+R	378.54	A I+R
f_{13}	569.58		487.57	A I+R	380.05	A I+R
f_{14}	744.50		508.31	A I+R	406.28	A I+R
f_{15}	755.39		512.49	A I+R	409.66	A I+R
f_{16}	766.06		682.96	A I+R	419.85	A I+R
f_{17}	975.24		772.93	A I+R	457.02	A I+R

f_{18}	986.44		777.37	A I+R	591.93	A I+R
f_{19}	1022.85		1047.92	A I+R	738.93	A I+R
f_{20}	1025.17		1050.32	A I+R	743.74	A I+R
f_{21}	1268.69	A" I+R	1284.07	A I+R	1062.62	A I+R
f_{22}			1286.94	A I+R	1070.44	A I+R
f_{23}			1386.01	A I+R	1351.72	A I+R
f_{24}			1463.49	A I+R	1355.90	A I+R
f_{25}					1584.62	A I+R
f_{26}					1591.94	A I+R
f_{27}					1677.30	A I+R

Table S14: Phonon frequencies of Mo-doped drum configurations. Infrared (I) and Raman (R) active modes are indicated.

Frequency	MoB_{16} (cm^{-1})	C_s	MoB_{18} (cm^{-1})	C_1	MoB_{20} (cm^{-1})	C_1
f_1	86.96	A' I+R	41.17	A I+R	106.48	A I+R
f_2	149.69	A' I+R	139.66	A I+R	137.41	A I+R
f_3	247.98	A' I+R	175.18	A I+R	163.80	A I+R
f_4	298.75	A" I+R	206.40	A I+R	183.62	A I+R
f_5	341.11	A' I+R	284.88	A I+R	192.86	A I+R
f_6	362.46	A" I+R	293.06	A I+R	199.49	A I+R
f_7	384.84	A' I+R	313.07	A I+R	243.12	A I+R
f_8	390.28	A" I+R	339.85	A I+R	251.63	A I+R
f_9	402.10	A' I+R	345.63	A I+R	256.67	A I+R
f_{10}	402.27	A" I+R	355.67	A I+R	321.15	A I+R
f_{11}	413.72	A" I+R	393.38	A I+R	326.87	A I+R
f_{12}	415.55	A' I+R	397.79	A I+R	343.29	A I+R
f_{13}	418.27	A" I+R	412.58	A I+R	359.64	A I+R
f_{14}	438.25	A' I+R	424.90	A I+R	363.27	A I+R

f_{15}	481.72	A" I+R	453.61	A I+R	386.09	A I+R
f_{16}	494.34	A' I+R	472.52	A I+R	399.55	A I+R
f_{17}	503.95	A' I+R	488.48	A I+R	423.97	A I+R
f_{18}	530.17	A" I+R	490.03	A I+R	451.92	A I+R
f_{19}	549.49	A" I+R	506.37	A I+R	461.98	A I+R
f_{20}	560.59	A" I+R	525.15	A I+R	463.18	A I+R
f_{21}	572.54	A' I+R	538.00	A I+R	464.44	A I+R
f_{22}	574.70	A' I+R	550.61	A I+R	488.79	A I+R
f_{23}	586.20	A' I+R	565.27	A I+R	490.98	A I+R
f_{24}	603.55	A' I+R	568.47	A I+R	494.90	A I+R
f_{25}	621.51	A" I+R	569.86	A I+R	499.06	A I+R
f_{26}	623.58	A" I+R	579.06	A I+R	501.98	A I+R
f_{27}	625.95	A' I+R	592.43	A I+R	503.32	A I+R
f_{28}	636.93	A" I+R	597.32	A I+R	503.87	A I+R
f_{29}	664.00	A' I+R	598.71	A I+R	520.83	A I+R
f_{30}	683.00	A' I+R	603.63	A I+R	523.70	A I+R
f_{31}	707.94	A' I+R	617.80	A I+R	536.05	A I+R
f_{32}	776.19	A' I+R	620.09	A I+R	539.91	A I+R
f_{33}	778.54	A" I+R	632.32	A I+R	547.71	A I+R
f_{34}	845.88	A" I+R	640.08	A I+R	550.01	A I+R
f_{35}	855.78	A' I+R	705.75	A I+R	552.95	A I+R
f_{36}	952.11	A' I+R	735.70	A I+R	559.29	A I+R
f_{37}	960.07	A" I+R	755.45	A I+R	561.40	A I+R
f_{38}	1046.70	A" I+R	823.60	A I+R	598.34	A I+R
f_{39}	1048.67	A' I+R	835.86	A I+R	652.15	A I+R
f_{40}	1057.82	A" I+R	969.00	A I+R	701.09	A I+R
f_{41}	1070.43	A' I+R	1006.34	A I+R	710.29	A I+R
f_{42}	1070.44	A" I+R	1039.57	A I+R	819.94	A I+R
f_{43}	1158.50	A' I+R	1056.87	A I+R	823.73	A I+R
f_{44}	1198.49	A" I+R	1191.11	A I+R	986.84	A I+R

f_{45}	1206.90	A' I+R	1208.72	A I+R	987.98	A I+R
f_{46}			1212.50	A I+R	1068.50	A I+R
f_{47}			1220.63	A I+R	1071.95	A I+R
f_{48}			1223.47	A I+R	1254.08	A I+R
f_{49}			1263.41	A I+R	1256.73	A I+R
f_{50}			1268.85	A I+R	1273.45	A I+R
f_{51}			1298.54	A I+R	1276.47	A I+R
f_{52}					1394.08	A I+R
f_{53}					1401.76	A I+R
f_{54}					1405.10	A I+R
f_{55}					1405.78	A I+R
f_{56}					1414.40	A I+R
f_{57}					1415.44	A I+R

SIII. LÖWDIN POPULATION ANALYSIS

The application of Löwdin population analysis, combined with the consideration of polarization effects, provided a comprehensive picture of the studied system's electronic structure. The Löwdin charge (q) and polarization (P) on each atom for all the studied clusters are given in Tables. from S15 to S21.

Table S15: Löwdin charge (q) and polarization (P) on each atom for Ti-doped boron clusters.

Cluster	TiB_8		TiB_9		TiB_{10}		TiB_{16}		TiB_{18}		TiB_{20}	
Atom	q	P	q	P	q	P	q	P	q	P	q	P
Ti	10.97	1.012	10.78	1.005	10.61	1.135	11.25	0	10.97	0.535	10.8	1.176
B	3.07	0.284	3.10	-0.003	3.08	0.011	2.98	0	2.98	0.095	2.98	0.017
B	3.07	0.284	3.08	-0.004	3.08	-0.126	2.98	0	3.01	-0.020	2.98	0.079
B	3.07	0.284	3.08	-0.004	3.08	-0.126	2.98	0	2.97	-0.031	2.98	-0.023
B	3.07	0.284	3.06	-0.003	3.08	0.011	2.98	0	2.98	0.087	2.98	0.041
B	3.07	0.284	3.06	-0.003	3.08	-0.048	2.98	0	3.00	0.077	2.98	0.103
B	3.07	0.285	3.07	-0.002	3.08	-0.048	2.98	0	2.98	0.009	2.98	-0.081
B	3.07	0.284	3.07	-0.002	3.08	-0.043	2.98	0	3.01	-0.006	2.98	0.119
B	3.07	0.284	3.09	-0.003	3.08	-0.124	2.98	0	2.98	0.029	2.98	-0.051
B			3.09	-0.003	3.08	-0.124	2.98	0	3.00	0.06	2.98	0.110
B					3.08	-0.043	2.98	0	2.99	0.097	2.98	-0.073
B							2.98	0	3.00	-0.029	3.01	0.078
B							2.98	0	3.00	-0.023	3.01	0.073
B							2.98	0	2.99	0.07	3.01	0.078
B							2.98	0	2.99	0.091	3.01	0.076
B							2.98	0	3.00	-0.01	3.01	0.088
B							2.98	0	3.00	0.015	3.01	-0.05
B									2.99	0.052	3.01	0.087
B									3.00	0.036	3.01	0.004
B											3.01	0.095

B											3.01	0.049

Table S16: Löwdin charge (q) and polarization (P) on each atom for Cr-doped boron clusters.

Cluster	CrB_8		CrB_9		CrB_{10}		CrB_{16}		CrB_{18}		CrB_{20}	
Atom	q	P	q	P	q	P	q	P	q	P	q	P
Cr	13.03	1.698	12.71	3.019	12.73	2.664	13.01	0	13.01	1.187	12.91	3.161
B	3.06	0.368	3.08	0.113	3.04	0.085	2.98	0	2.99	0.134	2.98	0.027
B	3.06	0.368	3.08	0.198	3.09	0.085	2.98	0	3.01	-0.018	2.96	0.027
B	3.06	0.368	3.08	0.198	3.09	0.085	2.98	0	2.97	0.039	2.99	0.026
B	3.06	0.368	3.08	0.319	3.04	0.085	2.98	0	2.99	0.132	2.97	0.026
B	3.06	0.368	3.08	0.319	3.06	0.085	3.00	0	3.00	0.126	2.93	0.027
B	3.06	0.368	3.08	0.271	3.06	0.085	3.00	0	2.98	0.090	2.99	0.027
B	3.06	0.368	3.08	0.271	3.06	0.085	2.99	0	3.00	-0.016	2.94	0.027
B	3.06	0.368	3.08	0.137	3.09	0.085	2.99	0	2.98	0.094	3.00	0.027
B			3.08	0.137	3.09	0.085	3.00	0	3.00	0.122	2.95	0.027
B					3.06	0.085	3.00	0	2.99	0.153	3.00	0.026
B							3.00	0	3.01	0.046	3.03	0.056
B							3.00	0	2.97	-0.022	3.04	0.056
B							3.00	0	2.98	0.114	3.05	0.055
B							3.00	0	2.99	0.152	3.01	0.055
B							3.00	0	2.97	-0.02	2.94	0.056
B							3.00	0	3.00	0.105	3.03	0.056
B									2.98	0.111	2.96	0.055
B									3.00	0.108	3.03	0.055
B											2.95	0.055
B											3.03	0.055

Table S17: Löwdin charge (q) and polarization (P) on each atom for Mn-doped boron clusters.

Cluster	MnB_8		MnB_9		MnB_{10}		MnB_{16}		MnB_{18}		MnB_{20}	
Atom	q	P	q	P	q	P	q	P	q	P	q	P
Mn	14.05	1.058	13.73	2.681	13.67	3.54	14.01	1.223	13.98	1.475	13.95	2.8323
B	3.06	0.24	3.08	-0.075	3.05	-0.097	2.99	-0.015	3.01	-0.028	3.03	0.010
B	3.06	0.241	3.08	-0.075	3.11	-0.187	2.99	-0.015	2.99	-0.027	2.95	0.081
B	3.06	0.24	3.08	-0.075	3.11	-0.187	2.99	-0.015	2.95	-0.02	3.02	-0.059
B	3.06	0.241	3.08	-0.075	3.05	-0.097	2.99	-0.015	3.00	-0.027	3.03	0.006
B	3.06	0.24	3.08	-0.075	3.06	0.078	3.01	-0.014	3.01	-0.029	2.94	0.105
B	3.06	0.24	3.08	-0.074	3.06	0.078	3.01	-0.014	2.97	-0.022	3.00	-0.093
B	3.06	0.24	3.08	-0.074	3.05	0.046	3.00	-0.011	2.99	-0.028	2.94	0.107
B	3.06	0.24	3.08	-0.075	3.10	-0.108	3.00	-0.011	2.98	-0.024	3.02	-0.065
B			3.08	-0.075	3.10	-0.108	2.99	-0.013	3.01	-0.029	2.95	0.08
B					3.05	0.046	3.00	-0.015	3.01	-0.029	3.00	-0.092
B							3.00	-0.015	2.99	-0.027	3.01	-0.011
B							2.99	-0.013	2.96	-0.02	2.98	0.043
B							3.00	-0.015	3.00	-0.026	3.01	-0.009
B							2.99	-0.013	3.01	-0.029	2.98	0.047
B							3.00	-0.015	2.96	-0.021	2.92	0.073
B							2.99	-0.013	3.00	-0.029	3.01	-0.108
B									2.99	-0.025	2.95	0.101
B									3.00	-0.029	3.01	-0.082
B											2.95	0.104
B											3.02	-0.085

Table S18: Löwdin charge (q) and polarization (P) on each atom for Fe-doped boron clusters.

Cluster	FeB_8		FeB_9		FeB_{10}		FeB_{16}		FeB_{18}		FeB_{20}	
Atom	q	P	q	P	q	P	q	P	q	P	q	P
Fe	14.88	2.051	14.81	1.744	14.73	2.702	14.98	0.913	14.9	1.518	15.09	1.389
B	3.08	-0.071	3.07	-0.081	3.07	-0.174	3.02	0.093	3.01	-0.009	3.05	-0.024
B	3.08	-0.071	3.07	-0.081	3.07	-0.168	3.02	0.093	2.99	0.121	3.01	0.045
B	3.08	-0.071	3.07	-0.081	3.07	-0.168	3.02	0.093	2.97	0.019	2.94	0.105
B	3.08	-0.071	3.07	-0.081	3.07	-0.174	3.02	0.093	3.00	-0.019	3.05	-0.027
B	3.08	-0.071	3.07	-0.081	3.06	0.060	2.97	-0.027	3.01	-0.006	2.99	-0.005
B	3.08	-0.07	3.07	-0.081	3.06	0.060	2.97	-0.027	2.97	0.017	2.94	-0.001
B	3.08	-0.071	3.07	-0.081	3.07	-0.025	2.97	-0.027	3.00	0.071	2.99	0.015
B	3.08	-0.071	3.07	-0.081	3.07	-0.035	2.97	-0.027	3.00	-0.018	2.93	0.113
B			3.07	-0.081	3.07	-0.036	3.00	0.038	3.00	0.065	3.00	0.055
B					3.07	-0.026	3.00	0.038	3.01	-0.023	2.94	0.019
B							3.00	0.038	2.99	0.107	3.00	0.003
B							3.00	0.038	2.97	0.026	2.99	0.055
B							3.00	0.038	3.00	-0.023	2.94	0.094
B							3.00	0.038	3.01	0.023	2.98	0.024
B							3.00	0.038	2.98	0.001	2.99	0.05
B							3.00	0.038	2.99	0.104	2.94	0.072
B									2.98	-0.003	3.00	-0.032
B									3.01	0.028	2.97	0.05
B											3.00	0.017
B											2.92	-0.024

Table S19: Löwdin charge (q) and polarization (P) on each atom for Co-doped boron clusters.

Cluster	CoB_8		CoB_9		CoB_{10}		CoB_{16}		CoB_{18}		CoB_{20}	
Atom	q	P	q	P	q	P	q	P	q	P	q	P
Co	16.00	-0.104	15.92	0	15.73	2.075	16	0.026	16.02	-0.002	16.03	0.872
B	3.06	-0.135	3.06	0	3.07	0.091	3.02	0.134	2.97	0.042	3.05	0.005
B	3.06	-0.135	3.06	0	3.07	0.092	3.02	0.134	3.00	0.007	3.03	-0.018
B	3.06	-0.136	3.06	0	3.07	0.091	3.02	0.134	3.00	0.01	2.95	-0.007
B	3.06	-0.136	3.06	0	3.07	0.092	3.02	0.134	2.98	0.14	3.05	0.005
B	3.06	-0.135	3.06	0	3.07	0.091	2.96	-0.02	2.98	0.132	2.99	0.061
B	3.06	-0.135	3.06	0	3.07	0.092	2.96	-0.02	3.00	0.067	2.94	-0.001
B	3.06	-0.136	3.06	0	3.07	0.092	2.96	-0.02	3.00	0.054	2.99	0.06
B	3.06	-0.136	3.06	0	3.07	0.091	2.96	-0.02	2.98	0.019	2.95	-0.007
B			3.06	0	3.07	0.092	2.99	0.064	2.98	0.023	3.03	-0.018
B					3.07	0.091	2.99	0.064	2.98	0.15	2.94	-0.001
B							2.99	0.064	3.00	0.023	2.99	-0.007
B							2.99	0.064	3.00	0.031	3.00	0.002
B							2.99	0.064	2.97	0.034	2.96	-0.009
B							2.99	0.064	2.97	0.037	2.96	-0.002
B							2.99	0.064	2.99	0.007	3.00	0.003
B							2.99	0.064	2.99	0.01	2.96	-0.009
B									2.99	0.108	2.98	0.068
B									2.99	0.095	2.96	-0.002
B											2.99	-0.007
B											2.90	0.011

Table S20: Löwdin charge (q) and polarization (P) on each atom for Nb-doped boron clusters.

Cluster	NbB_8		NbB_9		NbB_{10}		NbB_{16}		NbB_{18}		NbB_{20}	
Atom	q	P	q	P	q	P	q	P	q	P	q	P
Nb	12.73	0.249	12.53	-0.081	12.29	-0.033	12.84	-0.014	12.64	-0.017	12.44	0
B	2.97	0.524	3.00	0.229	3.02	0.103	2.94	0.005	2.95	0.118	2.97	0
B	2.97	0.524	3.00	0.229	3.02	0.103	2.94	0.005	2.97	-0.014	2.97	0
B	2.97	0.524	3.00	0.229	3.02	0.103	2.94	0.005	2.97	0.013	2.97	0
B	2.97	0.524	3.00	0.229	3.02	0.103	2.94	0.005	2.96	0.069	2.97	0
B	2.99	0.158	3.00	0.229	3.02	0.100	2.96	0.09	2.95	0.132	2.97	0
B	2.99	0.158	3.00	0.228	3.02	0.101	2.96	0.09	2.97	-0.013	2.97	0
B	2.99	0.158	3.00	0.228	3.02	0.101	2.97	0.15	2.96	0.067	2.97	0
B	2.99	0.158	3.00	0.228	3.02	0.101	2.97	0.15	2.95	0.107	2.97	0
B			3.00	0.228	3.02	0.101	2.95	0.085	2.96	0.024	2.97	0
B					3.02	0.101	2.95	0.042	2.95	0.113	2.97	0
B							2.95	0.042	2.96	0.017	2.97	0
B							2.95	0.085	2.97	-0.015	2.97	0
B							2.95	0.042	2.95	0.131	2.97	0
B							2.95	0.085	2.96	0.075	2.97	0
B							2.95	0.042	2.96	0.06	2.97	0
B							2.95	0.085	2.97	-0.011	2.97	0
B									2.96	0.02	2.97	0
B									2.95	0.113	2.97	0
B											2.97	0
B											2.97	0

Table S21: Löwdin charge (q) and polarization (P) on each atom for Mo-doped boron clusters.

Cluster	MoB_8		MoB_9		MoB_{10}		MoB_{16}		MoB_{18}		MoB_{20}	
Atom	q	P	q	P	q	P	q	P	q	P	q	P
Mo	13.09	-0.174	13.05	0.712	12.83	0.674	13.07	0	12.96	0	12.94	0
B	3.06	0.269	3.05	0.190	3.07	-0.033	3.00	0	3.01	0	2.98	0
B	3.06	0.269	3.05	0.190	3.06	-0.049	3.00	0	3.00	0	2.98	0
B	3.06	0.269	3.05	0.190	3.06	-0.035	3.00	0	2.98	0	2.98	0
B	3.06	0.269	3.05	0.190	3.06	-0.064	3.00	0	3.02	0	2.98	0
B	3.06	0.270	3.05	0.190	3.04	-0.14	3.00	0	3.00	0	2.98	0
B	3.06	0.270	3.05	0.190	3.07	-0.046	3.00	0	3.01	0	2.98	0
B	3.06	0.270	3.05	0.190	3.05	-0.099	3.01	0	2.98	0	2.98	0
B	3.06	0.270	3.05	0.189	3.07	-0.041	3.01	0	2.98	0	2.98	0
B			3.05	0.189	3.05	-0.119	2.98	0	3.03	0	2.98	0
B					3.07	-0.037	2.98	0	3.00	0	2.98	0
B							2.98	0	2.98	0	3.00	0
B							2.98	0	2.97	0	3.00	0
B							2.98	0	2.97	0	3.00	0
B							2.98	0	3.00	0	3.00	0
B							2.98	0	2.95	0	3.00	0
B							2.98	0	3.00	0	3.00	0
B									3.01	0	3.00	0
B									2.97	0	3.00	0
B											3.00	0
B											3.00	0
